# Metabolic Profiling of PGPR-Treated Tomato Plants Reveal Priming-Related Adaptations of Secondary Metabolites and Aromatic Amino Acids

**DOI:** 10.3390/metabo10050210

**Published:** 2020-05-20

**Authors:** Msizi I. Mhlongo, Lizelle A. Piater, Paul A. Steenkamp, Nico Labuschagne, Ian A. Dubery

**Affiliations:** 1 Department of Biochemistry, University of Johannesburg, Auckland Park, Johannesburg 2006, South Africa; mmhlongo@uj.ac.za (M.I.M.); lpiater@uj.ac.za (L.A.P.); psteenkamp@uj.ac.za (P.A.S.); 2 Department of Plant and Soil Sciences, University of Pretoria, Hatfield, Pretoria 0028, South Africa; nico.labuschagne@up.ac.za

**Keywords:** chemometrics, metabolomics, metabolic reprogramming, plant defense, plant growth-promoting rhizobacteria, priming

## Abstract

Plant growth–promoting rhizobacteria (PGPR) are beneficial microbes in the rhizosphere that can directly or indirectly stimulate plant growth. In addition, some can prime plants for enhanced defense against a broad range of pathogens and insect herbivores. In this study, four PGPR strains (*Pseudomonas fluorescens* N04, *P. koreensis* N19, *Paenibacillus alvei* T19, and *Lysinibacillus sphaericus* T22) were used to induce priming in *Solanum lycopersicum* (cv. Moneymaker) plants. Plants were inoculated with each of the four PGPRs, and plant tissues (roots, stems, and leaves) were harvested at 24 h and 48 h post-inoculation. Methanol-extracted metabolites were analyzed by ultra-high performance liquid chromatography mass spectrometry (UHPLC-MS). Chemometric methods were applied to mine the data and characterize the differential metabolic profiles induced by the PGPR. The results revealed that all four strains induced defense-related metabolic reprogramming in the plants, characterized by dynamic changes to the metabolomes involving hydroxycinnamates, benzoates, flavonoids, and glycoalkaloids. In addition, targeted analysis of aromatic amino acids indicated differential quantitative increases or decreases over a two-day period in response to the four PGPR strains. The metabolic alterations point to an altered or preconditioned state that renders the plants primed for enhanced defense responses. The results contribute to ongoing efforts in investigating and unraveling the biochemical processes that define the PGPR priming phenomenon.

## 1. Introduction 

Agricultural food production is hampered by a plethora of plant pathogens and herbivores that lower crop yields [1,2,3]. To obviate this problem, modern cultivation processes rely on breeding for resistance and extensive use of agrochemicals that pose human [4] and environmental hazards [5,6]. Hence, there is a need for new environmentally friendly ways to minimize the negative effects of both pathogens and herbivores. In this regard, the rhizosphere-inhabiting microorganisms are being investigated for improving plant health and growth without extensive use of agrochemicals [7,8,9,10]. 

The rhizosphere is the microscopic zone surrounding plant roots in which a complex relationship co-exists between a plant, soil microbes, and the soil itself. This zone harbors both deleterious (pathogens) and beneficial organisms (symbiotic rhizobia, certain actinomycetes, mycorrhizal fungi, and free-living bacteria) that have either negative or positive effects on plant growth and production [7,11]. Plant growth–promoting rhizobacteria (PGPRs) are free-living bacteria inhabiting this rhizosphere, and these organisms can either directly (through nutrient solubilization, nitrogen fixation and production of phytohormones and siderophores) or indirectly (through production of antibiotics, hydrolytic enzymes, and exo-polysaccharides and priming or induction of resistance), improve plant health and prime plants against a broad spectrum of pathogens [7,8,9]. Plant–microbe interactions with beneficial organisms such as PGPRs are symbiotic, in which fitness costs and benefits are shared by the interacting organisms [12]. For instance, plants secrete root exudates that are selective for certain strains of bacterial communities, and in return, the latter secrete metabolites beneficial to the plant [10,13,14].

Rhizosphere-inhabiting microorganisms such as PGPRs have been extensively studied for their beneficial properties and reports show that they possess a variety of qualities such as plant growth promotion, disease control and bioremediation [8,15,16]. PGPRs have been shown to directly/indirectly reduce impact of environmental stresses by rendering plants more resistant to environmental stresses (both biotic and abiotic) as well as suppress soil-borne pathogens and herbivores through secretion of antibiotics, toxins, lytic enzymes, and siderophores [15,17]. Plant priming is defined as induced readiness to secondary stresses, thus increasing plant survival. However, priming success depends on the timing, strength, and specificity of the activated immune responses [18,19,20,21,22,23,24,25]. Previous studies have shown that inoculation of plants with PGPRs or PGPR exometabolites for the purpose of enhancing defense responses, lead to various biochemical events associated with cell wall modification, expression of defense genes, primary metabolite modification, and biosynthesis of secondary metabolites [18].

In order to gain a deeper understanding of the biochemical events associated with the early stage of PGPR priming or PGPR-induced resistance, the objective of this investigation was to profile perburbations to the metabolomes of roots, stems and leaves, as well as to characterize the metabolic biomarkers in tomato plants responding to PGPRs. Thus, untargeted metabolic profiling based on ultra-high performance liquid chromatography mass spectrometry (UHPLC-MS) metabolomics was used in combination with multivariate statistical analyses to study the metabolic changes induced by four PGRP strains (species belonging to the *Pseudomonas* (2)*, Lysinibacillus* (1), and *Paenibacillus* (1) genera). Furthermore, targeted analysis of aromatic amino acids feeding into secondary metabolism (mostly to the phenylpropanoid pathway) was performed because of the importance of this pathway in plant–microbe interactions and plant adaptation to ever-changing environments. 

## 2. Results 

### 2.1. Aromatic Amino Acid Quantification in Tomato Plant Tissues

**Figure 1:**
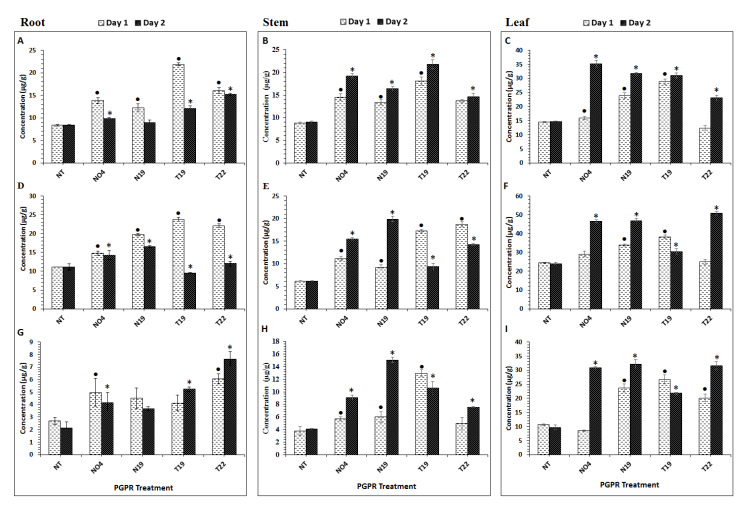
Plant growth–promoting rhizobacteria (PGPR)-induced changes in aromatic amino acid concentrations in tomato plant tissues. (**A**–**C**): Phenylalanine, (**D**–**F**): Tryptophan, (**G**–**I**): Tyrosine. The values are means ± SD (*n* = 3 independent samples). Extracts were prepared from 200 mg of root, stem and leaf tissue, and all concentrations are expressed as µg/g fresh weight (FW). NO4= *Pseudomonus fluorescens*; N19 = *Pseudomonus koreensis*; T19 = *Paenibacillus alvei*; T22 *= Lysinibacillus sphaericus*. An asterix (*) or a dot (•) indicates the statistical significance with a *p*-value < 0.05 compared with the non-treated (NT) plants. • = comparison on day 1 post-inoculation; * = comparison on day 2 post-inoculation.

In order to investigate PGPR influence on the primary metabolism of tomato plants, 50% methanol extracts prepared from tissues at day 1 and 2 post-inoculation, were analyzed. Aromatic amino acids (Phe, Trp, and Tyr) were quantified using a multiple reaction monitoring (MRM) method established on a triple quadrupole mass spectrometer(QqQ-MS) instrument. The data is shown in Figure 1, where quantitative values from treated plants are compared to those from non-treated (NT) plants at days 1 and 2. Phe (Figure 1A–C) was found to be differentially modulated in the different plant tissues by the various PGPR inoculations. In the inoculated roots (Figure 1A), the Phe content was significantly up-regulated by the four PGPRs (day 1), with T19 showing the most significant increase when compared to the other strains, with an overall, slight decrease on day 2. The opposite was observed in both stems (Figure 1B) and leaves (Figure 1C), where Phe had a significant increase in concentration on day 1 and continuing to increase on day 2, except for leaf tissue from T22-inoculated plants, where a decrease was observed on day 2 (however, not significant when compared to the control (Figure 1C)). 

The Trp content was significantly higher on day 1 in roots treated with *P. fluorescens* N04 (Figure 1D) and differentially regulated between days 1 and 2. Results for stem (Figure 1E) and leaf (Figure 1F) samples showed that Trp was differentially regulated in these tissues as well. The Trp content in the stems displayed a significant increase from day 1 to day 2 in the *Pseudomonas* (N19 and N04)-inoculated plants, whereas the *Paenibacillus* (T19) and *Lysinibacillus* (T22)-inoculated plants showed a significant accumulation of Trp on day 1, followed by a decrease thereafter, but remaining higher than the control (Figure 1). The same was observed in the leaf samples, with *Pseudomonas*-inoculated (N04 and N19) plants showing a gradual increase from day 1 to 2 (Figure 1F). The T22 *Lysinibacillus*-treated plants displayed a significantly higher Trp content on day 2 compared to day 1 (Figure 1F). 

The Tyr content was found to be significantly higher on day 1 in roots (Figure 1G); however, this changed from day 1 to day 2. N04-treated plants showed a significant increase in roots on day 1 but decreased on day 2, whereas no significant difference between control plants and N19-treated plants were found. For *Lysinibacillus*- (T19) and *Paenibacillus* (T22)-treated plants, accumulation of Tyr from day 1 to 2 was observed in the roots (Figure 1G). Stem (Figure 1H) and leaf (Figure 1I) samples generally showed that Tyr continued to increase significantly from day 1 to 2 with the exception of T19 inoculated plants, where Tyr accumulated on day 1 and decreased on day 2, but it still remained significantly higher than in the controls (Figure 1H,I). 

### 2.2. Metabolic Profiling of PGPR-Induced Changes in Tomato Tissues

**Figure 2:**
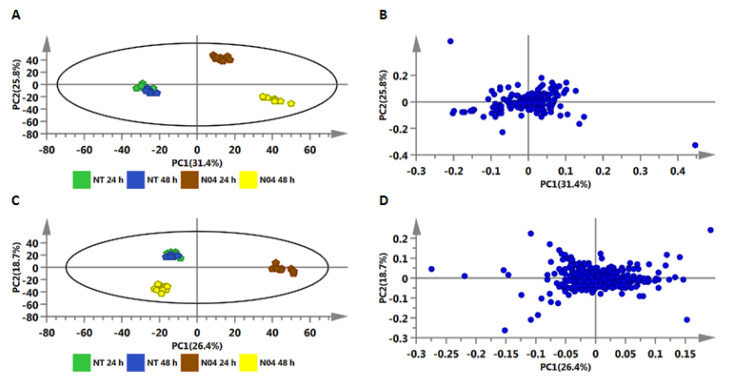
The principal component analysis (PCA) scores and loadings plots of metabolites extracted from tomato plant stems treated with *Pseudomonas fluorescens* N04. The PCA scores scatter plots (**A** and **C**) show clear separation and grouping of control and treated samples, respectively. The PCA loadings plots (**B** and **D**) show ions contributing to the clustering. The X-axis and Y-axis describe the first and second principle components (PCs), respectively. (**A** and **B**) = ESI negative data and (**C** and **D**) = ESI positive data.

Untargeted metabolomics was used to profile metabolic changes in tomato root, stem, and leaf tissues treated with the two *Pseudomonas* (N04 and N19), *Lysinibacillus* (T19), and *Paenibacillus* (T22) spp. at 24 h and 48 h post-inoculation. The experiments consisted of three independent biological replicates, and the samples were analyzed in triplicate. Principal component analysis (PCA) uncovered hidden internal structures of the data in two dimensions: the first two principal components (PCs) revealed time-dependent and inoculation-related clustering of samples (Figure 2A,C). PCA loading plots (Figure 2B,D) allowed the identification of features related to the observed clustering (by superimposing the PCA loadings plot, one can identify some features correlated to the clustering). These PCA-selected metabolic features were confirmed and complemented by orthogonal projection to latent structures discriminant analysis (OPLS-DA) modeling, a binary-classification method, allowing a discrimination analysis to extract metabolic markers that differentiate sample groups depicted by PCA models [26,27,28]. Figure 2 corresponds to data (from both positive and negative MS modes) of stem extracts from plants treated with *Pseudomonas fluorescens* N04. The equivalent sets of figures for extracts prepared from leaves, stems, and roots for all four different PGPR treatments are presented as supplementary data in Figures S3–S13A,C (PCA scores plots) and Figures S3–S13B,D (PCA loadings plots), respectively. 

On the PCA scores plots, the quality control (QC) samples were found to cluster tightly together toward the center (not shown), thus confirming the UHPLC-MS stability, analysis reliability, and reproducibility with minimal variation. 

**Figure 3:**
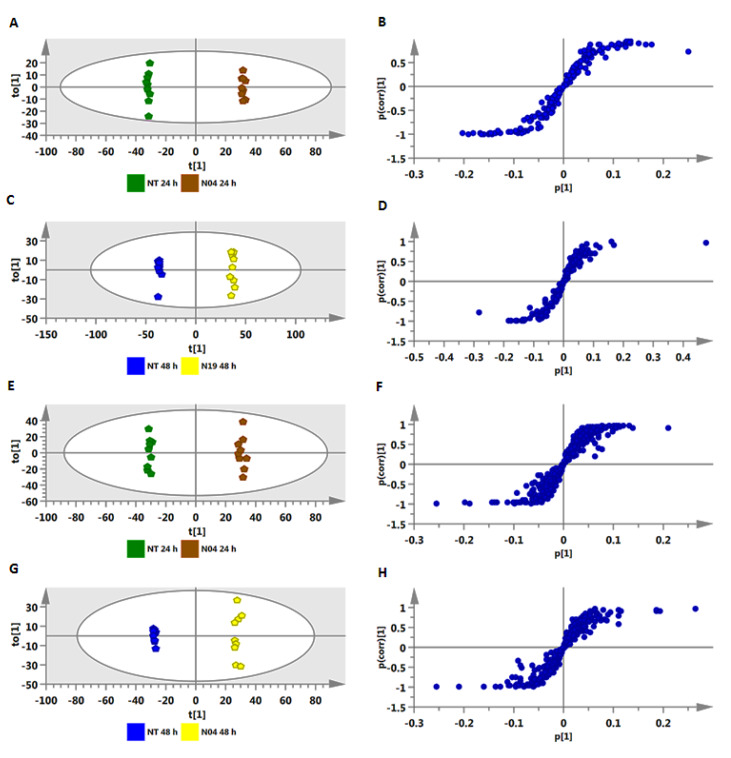
Identification of discriminatory biomarkers present in methanolic extracts of stem tissue of tomato plants treated with *Pseudomonas fluorescens* N04, using orthogonal projection to latent structures discriminant analysis (OPLS-DA) modeling. The OPLS-DA score plots (**A, C, E**, and **G**) and the corresponding S-plots (**B, D, F**, and **H**) show the different clustering of treated and non-treated samples (NT 24 h vs. T 24 h and NT 48 h vs. T 48 h). The ellipse represents Hotelling’s T^2^ with 95% confidence. Model validation by cross-validated analysis of variance (CV-ANOVA) showed high model significance with a *p*-value ˂ 0.05. (**A**–**D**) = ESI negative data and (**E**–**H**) = ESI positive data.

The OPLS-DA models were computed using a combination of two predefined conditions, non-treated (NT) 24 h vs. treated (T) 24 h and NT 48 h vs. T 48 h. The OPLS-DA score plots (NT 24 h vs. T 24 h and NT 48 h vs. T 48 h) (Figure 3A,C,E,G) show a clear separation of the treated from control samples. The corresponding OPLS-DA loading S-plots (Figure 3B,D,F,H) highlighted relevant significant ions with both high covariance and correlation. These signatory/discriminatory markers are the “outlier” ions in the upper right quadrant (positively correlated to the treatment), and those in the lower left quadrant (negatively correlated to the treatment). As mentioned in the experimental procedures (Section 4.6), correlation [*(p(corr)*] ≥ |0.6| and covariance *(p1)* ≥ |0.05| were chosen as cut-off values for selection of discriminating ions. The latter were further validated via variable importance (influence on) in projection (VIP) scoring, a metric that summarizes the importance of each variable in driving the observed group separation in a classification model. A variable with VIP score > 1.0 means that the variable contributes more than average to the classification model [26]. The equivalent sets of figures for extracts prepared from leaves, stems and roots (NT 48 h vs. T 48 h) are presented as supplementary data in Figures S14–S24A,C,E,G (OPLS-DA scores plots) and Figures S14–S24B,D,F,H (OPLS-DA S plots), respectively. 

#### 2.2.1. Annotation of Signatory Biomarkers of PGPR Priming

**Figure 4:**
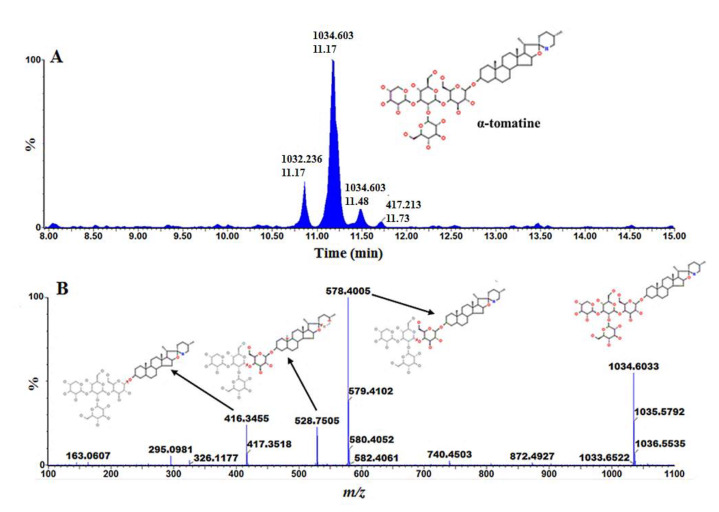
Representative UHPLC-MS/MS single ion chromatogram (XIC) and spectrum of α-tomatine. (**A**) single ion chromatograms (XIC) of UHPLC-MS/MS showing the retention time of α-tomatine. (**B**) MS spectra showing fragmentation patterns of α-tomatine. Annotation of significant ions from the treated tomato plants was performed with the aid of UHPLC-qTOF-MS/MS, based on the “in-source collision-induced dissociation” (ISCID) method as previously reported [31,37]. Single ion chromatograms were extracted and the spectral fragments compared among the different collision energies and with available spectral information. The mass spectrum of the extracted ion peak was used to deduce the putative empirical formula of the compound.

Due to the diverse physicochemical properties of plant metabolites, metabolite annotation is still a daunting task. Moreover, the limitation of databases and the availability of authentic standards contribute to the possible incorrect annotation of metabolites. Recently, metabolomics has seen much-needed instrument improvements with mass accuracy below 3 ppm and high-resolution [29]. These improvements permit the unambiguous determination of an empirical formula that can be used to search various databases. Advanced instruments are equipped with a high scanning power and the ability to acquire un-fragmented and fragmented data in a single run by ramping the in-source collision energy. To correctly annotate the discriminant ions, various factors from the chromatographic separation to MS were taken into consideration as reported previously [30,31,32,33,34,35]. In the present study, procedures involving tandem MS (MS/MS) for compound identification [31,32,34,35,36,37] were adopted. This involved comparison of MS spectra to published data, using online databases to search for matches to the generated empirical formulae. Table 1 summarizes the annotated discriminant ions/features (at metabolomics standards initiative (MSI) level-2 annotation) [36]. Shown below are the single ion chromatograms (XIC, Figure 4A) and associated MS spectra (Figure 4B) for α-tomatine observed to be differentially regulated by the different strains.

#### 2.2.2. Heat Map Analysis

**Figure 5:**
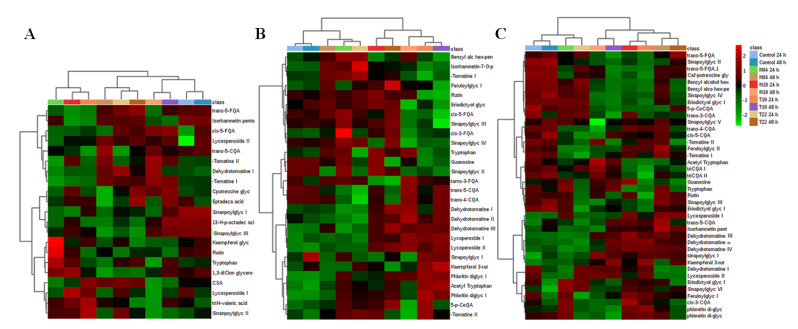
Heat map and hydroxycinnamic acid (HCA) dendrogram representing sample similarities for (**A**) roots, (**B**) stems, and (**C**) leaves. The metabolites are indicated in rows, and the 24 h and 48 h data for PGPR-inoculated tomato plant samples are shown in columns together with the control. Columns are *Pareto* scaled, with relative abundance represented by color (green lower abundance; red, higher abundance), as indicated in the key. Color brightness corresponds to the magnitude of the difference when compared with the average value. Analysis of the heat maps show plant tissue and PGPR strain–specific metabolic changes. Dendrograms are shown on top and to the left of the heat maps. In this instance, dendrograms are used to show hierarchical relationships between conditions/inoculations (top) and identified metabolites (left). The dendrograms show two main groups of clustering: the first one is associated with the control or responses similar to the controls, the second cluster is highly distinct from the control, indicating different metabolic reprogramming. (Produced on the Metaboanalyst web, [40]).

Following metabolite annotation, peak intensities were used to construct heat maps illustrating differences in the relative concentrations in the various tissues, responding to PGPR inoculation (Figure 5). Heat maps allow the visualization of large multidimensional data sets, and identify metabolic trends and patterns under similar or different experimental conditions. In addition, heat maps can be used to locate hidden groups among identified metabolites and associations between experimental groups and metabolic changes. Incorporation of hierarchical clustering (on row and columns) allows the grouping of similar patterns [38,39,40]. Thus, heat maps can be used to find specific sample groupings and associations with annotated metabolites. Figure 5 shows heat maps constructed from tissue of non-inoculated plants (NT) and inoculated plants (N04, N19, T19, and T22), harvested at 24 h and 48 h post-inoculation. 

Data are displayed in a grid where rows represent metabolites and columns represent the different inoculation and time points. The color and intensity of the cells represent metabolic regulation (up/down) in response to PGPR inoculation. Metabolites that showed significant increases are displayed in red, while significantly decreased metabolites are shown in green. Black represents an unchanged condition. On both sides (top and left) two main clusters are seen, which are then further subdivided to smaller clusters. These clusters show the relationship between condition and the identified metabolite content/concentration in response to inoculation with the PGPR strains. For example, in Figure 5C, the top dendrograms show that the control, N04, and T22 24-h inoculations lead to similar metabolic pertubations, whereas the opposite is true for the other conditions.

## 3. Discussion

Priming can be considered a highly adaptive trait where a retained memory of a prior stress event allows for accelerated and amplified responses, metabolically and physiologically, to subsequent events. In the case of defense priming, plants adjust their vulnerability to environmental stresses and deploy the defense response network in a faster, stronger and sustained manner following signal perception [15,18,19]. It is likely that agro-economically important PGPR will exhibit different mechanisms of action [8,15] and thus elicit differential effects on plant metabolism as investigated here. However, bacterial growth rates and viability in the sand medium as well as root adherence post-inoculation were not investigated, and these aspects could have a bearing on the differential temporal changes observed in the metabolomic reprogramming. 

### 3.1. Roles of Aromatic Amino Acids in PGPR Priming

Aromatic amino acids have a common chorismic acid precursor and play important roles in plant responses towards environmental perturbations. Although part of primary metabolism, these amino acids link to secondary metabolism, supplying precursors or carbon skeletons for the synthesis of secondary metabolites. The selected PGPR strains were found to differentially regulate Phe concentrations within the tomato tissues subsequent to root inoculation. Relatedly, inoculation of *Arabidopsis thaliana* roots with *Pseudomonas* sp. GM30 (isolated from poplar rhizosphere samples) was shown to lead to high abundance of Phe [41]. This amino acid is the entry point to the phenylpropanoid pathway that plays an important role in plant–pathogen interactions and plant priming. It includes the production of monolignols for cell wall lignification that provides mechanical resistance to pathogens. In addition to cell wall enforcement, phenylpropanoid products such as flavonoids and hydroxycinnamic acid (HCA) derivatives are actively involved in chemical defense [33,37,41,42,43].

Tyr and Phe are the products of the shikimate pathway and share prephenate/arogenate as precursor molecules. Both amino acids feed into secondary metabolism but may be utilized differentially by downstream networks of secondary metabolism, depending on the metabolic pathways active in the tissue types. Similar to Phe, Tyr is an important precursor in plant defense responses against pathogens, leading to phenol biosynthesis and lignin accumulation [44,45]. In addition, Tyr is also the precursor for tyramine biosynthesis [45]. Tyramines conjugated to HCA derivatives were found to accumulate in *Nicotiana tabacum* cells treated with various priming agents (both chemical and pathogen-derived) [36,37]. 

Trp modulation during plant-pathogen interactions has been reported. Indole derivatives, for example, are well-documented plant defense metabolites and these are derived from Trp in *A. thaliana* [41]. Plants of *A. thaliana* elicited with bacterial lipopolysaccharides accumulated a spectrum of Trp-derived indoles, including indole glucosinolates and indole acetic acid [46,47]. It was also reported that tomato plants accumulated Trp and methyl-Trp when challenged by *Botrytis cinerea* and *P. syringae*, and this was associated with the accumulation of the Trp derivative indole-carboxylic acid [41]. 

### 3.2. Metabolic Profiling of PGPR-Inoculated Tomato Plants

The link between primary and secondary plant metabolism plays a pivotal role in mediating plant–microbe interactions. These can occur with mutualistic, pathogenic, parasitic or neutral microbes which may have differential effects (positive or negative) on plant growth and development. Plant interactions with microbial organisms lead to changes in primary metabolism (mostly for energy production and biosynthesis of precursors for secondary metabolism) and modification of secondary metabolism (e.g., biosynthesis of defense-related metabolites with antimicrobial properties and cell wall lignification by phenolic precursors) [34,36,42,43]. UHPLC-MS metabolic profiling highlighted dynamic metabolic changes in PGPR-inoculated vs. non-inoculated tomato plants, and these changes were found to be strain-specific. In addition, this metabolic reprogramming was characterized by amino acids, organic acids, HCA derivatives, hydroxybenzoic acids, fatty acids, and glycoalkaloids (Table 1). In previous studies, inoculation of rice and maize with *Azospirillum* species was reported to affect HCAs and benzoxazines, respectively, and strain-specific metabolic changes were observed [48,49]. Also, using different microbial-derived priming agents, [37] showed that lipopolysaccharides, chitosan, and flagellin petide (flg22) triggered the biosynthesis of HCAs, and these were specific to each priming agent. Thus, the results indicate that plants use similar metabolites to interact with microbes but in different ways. The composition of the secondary metabolites in the altered plant metabolome and the concentrations of individual metabolites comprising these “chemical cocktails” are often determining factors in the quantitative phenotypic response of plants or cultivars to pathogenic organisms [34,35].

#### 3.2.1. Tryptophan and Its N-Acetyl Derivative as Markers of PGPR Priming

Trp and N-acetyl Trp were annotated and found to be differentially reprogrammed in the various tissues by the different PGPR strains (Figure 5). Particularly, only Trp was annotated in root and leaf tissue (Figure 5A,C)++99999996hereas N-acetyl Trp was only found in stems and leaves (Figure 5B,C). While the metabolomics data cannot distinguish between possible uptake of indole-like substrates from microbial-borne resources or endogeneous synthesis through the shikimate pathway, the latter should be the main contributor to the Trp pool. Amino acids play various roles in plant growth and development; however, during plant-microbe interactions, they could act as immediate suppliers of carbon and nitrogen required for the synthesis of defense-related metabolites [33,50]. For example, Trp is converted to serotonin involved in defense responses in rice against *Bipolaris oryzae* [51]. Relatedly, amino acid acetylation was reported as the regulatory hub for plant defense [52,53].

#### 3.2.2. Guanosine as a Marker of PGPR Priming

Guanosine was identified as differentially regulated in extracts from stems and leaf tissue by the different strains (Figure 5B,C). This molecule is a purine nucleoside comprising guanine attached to a ribose ring via a β-N₉-glycosidic bond. It has been reported to play a role in plant defense regulation by phosphorylation/dephosphorylation signaling mechanisms [53]. Guanosine tetraphosphate was reported to regulate salicylic acid signaling and *A. thaliana* resistance to *Turnip mosaic* virus (TuMV-GFP) [54]. Here, it was observed that high levels of guanosine tetraphosphate reduced SA levels and down-regulated numerous defense-related genes, whereas lower levels had an inverse effect, with increased resistance to TuMV-GFP. Based on these observations, the authors proposed that guanosine tetraphosphate could function at an early stage of infection. 

#### 3.2.3. Phenylpropanoid Pathway Metabolites as Markers of PGPR Priming

**Figure 6:**
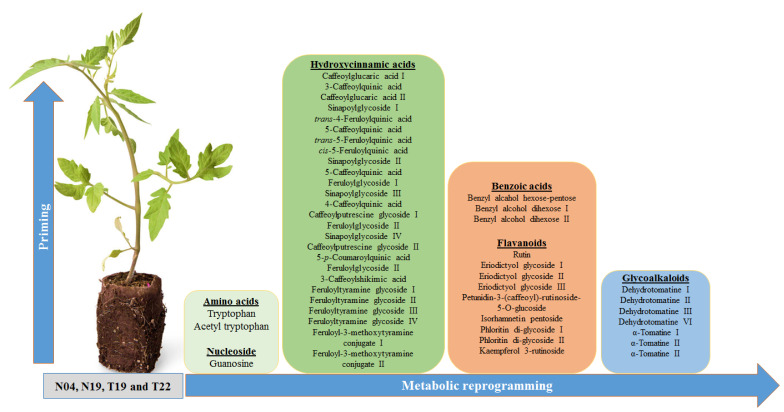
Signatory biomarkers induced by PGPR inoculation of tomato plants. A total of 48 metabolites were found to be differentially reprogrammed due to root inoculation by the different PGPR (N04, N19, T19, and T22). They belong to the metabolite classes of amino acids, nucleosides, various derivatives of hydroxycinnamic acids and benzoic acids, flavonoids and steroidal glycoalkaloids. Even though the plants were inoculated with different PGPR strains, the inoculation lead to activation of similar pathways, but with different metabolic profiles.

Phenolic molecules produced by the phenylpropanoid pathway play various functions during plant development and confer resistance to environmental stresses. This pathway drives the carbon flow from Phe/ Tyr in some instances for the production of 4-coumaroyl-CoA. This metabolite can then be converted to chalcone (for the production of flavonoids) [55], caffeoyl-CoA (for the production of HCA derivatives) [33,56], and coumaroylaldehyde (for production of monolignols) (Figure 6) [55]. In addition, 4-coumaroyl-CoA is a precursor for benzoic acid production via the β-oxidative or non-β-oxidative pathway [57,58]. These molecules strengthen the cell wall via lignification and can directly or indirectly inhibit pathogen progression, thus conferring resistance. Moreover, the phenylpropanoid products can be conjugated to various molecules such as organic acids, sugars, polyamines and amino acids [34,35,36,42]. In this study, three classes of molecules, namely benzoic acids, HCA derivatives, and flavonoids, derived from the phenylpropanoid pathway were annotated (Figure S25). 

Benzoic acids were detected in stem (Figure 5B) and leaf tissue (Figure 5C) of PGPR-treated plants. Only one benzoic acid derivative (benzyl alcohol hexose-pentose) (Figure 5B) was found in stem tissue, and three (benzyl alcohol hexose, benzyl alcohol hexose-pentose, and benzyl alcohol O-[α-_L_-arabinofuranosyl-(1,6)-β-_D_-glucopyaranoside]) were found in leaf tissue (Figure 5C). These molecules showed a PGPR strain-specific perturbation or either high or low content post-inoculation (Figure 5B,C). Benzoic acid and its derivatives are incorporated into different molecules, thus playing various roles in plant development and fitness (growth regulation, resistance, and attraction of pollinators) [57,58]. Tomato plants are known to produce a number of phenolic molecules in response to pathogen perception. For example, tomato plants infected with *Ralstonia solanacearum* [34] and tomato curly stunt virus (ToCSV) [59] showed a high increase in phenolic molecules, and some were annotated as benzoic acid derivatives. Moreover, indole-3-acetic acid application to tomato roots reduced *Fusarium oxysporum* f.sp. *lycopersici* infection and benzoic acids were among the secondary metabolites detected [60].

Flavonoids are well-documented secondary metabolites that confer resistance against various plant pathogens [42]. They were annotated in the tissues under investigation (Table 1) and were found to be differentially regulated by the four PGPR strains tested (Figure 5). Flavonoids can act specifically (by inducing the hypersensitive response in infected cells and the direct inhibition of pathogen enzymes) or non-specifically (antioxidant properties and quenching of reactive oxygen species generated by infection or in response to infection) in conferring resistance [61,62,63,64]. Moreover, these molecules can contribute to cell wall strengthening by modulating indole-3-acetic acid, causing tissue differentiation, tylose and callus formation, and closure of the vascular system to prevent both infection and to limit pathogens spreading [64,65]. In addition, nonpolar flavonoids have the ability to disrupt pathogens’ membranes and respiration. The B ring of flavonoids may also inhibit pathogens’ gene expression or DNA/RNA synthesis by forming hydrogen bonds with nucleic acid bases and can also inhibit polymerases [66]. In barley mutants (*ant* 18-159), proanthocyanidins or low concentrations of dihydroquercetin were found to confer protection against *Fusarium* spp. Furthermore, flavonoids were accumulated in varying levels in sorghum plants in response to bacterial and fungal infection [42,43].

Lastly, several HCA derivatives were annotated (Table 1). These molecules were found to be conjugated to sugar, quinic acid, polyamines, and glycerol and showed tissue specificity (present or absent in some tissues) and PGPR strain-specific reprogramming (Figure 5). HCA derivatives have been identified as phytoanticipins [67] and biomarkers conferring resistance to a wide variety of environmental stresses [34,68,69]. These molecules can act on pathogens either directly (inhibition of pathogen-associated enzymes by binding to amino and sulfhydryl groups of proteins) or indirectly (anti-oxidant properties) [70,71]. Previous priming studies in pepper [72] and *N. tabacum* cells [36,73] demonstrated that microbial-derived priming agents induced dynamic changes associated with HCA derivatives conjugated to quinic acid, sugars, shikimic acid, and polyamines. In addition, the perturbation of the metabolic pools of chlorogenic acids appeared dependent on the type of priming agent [36]. 

The priming mechanism in Solanaceous plants is known to involve the phenylpropanoid pathway leading to the accumulation of free and conjugated HCAs [33,36,67,73]. When the plant is infected by a pathogen, these conjugated HCAs are hydrolyzed by various enzymes to the HCA derivatives (p-coumaric, caffeic, ferulic, and sinapic acids) that are utilized in chemical defense pathways such as phytoalexin synthesis or cell wall strengthening. Thus, this interconversion of conjugated HCAs to free HCAs explains the accumulation of ester and amide conjugates of HCAs during priming [33]. Plants that have been primed to accumulate high levels of these HCA conjugates are enabled to launch a stronger and faster defense response upon subsequent infections [36,73].

#### 3.2.4. Fatty Acids as Markers of PGPR Priming

Three fatty acids were annotated in root tissue only (Figure 5A), including heptadecanoic acid, 13-hydroperoxyoctadecadienioc acid, and trihydroxyvaleric acid (Table 1). Differential reprogramming of these molecules by PGPR inoculation was observed and shown to be time-dependent. The evidence of fatty acid involvement in plant defense responses is not clear, but more studies reporting fatty acid identification in response to pathogens are emerging. For example, in sorghum plants infected with bacterial and fungal pathogens, fatty acids were identified as signatory biomarkers [42,74]. Moreover, *Paenibacillus alvei* (T22)-primed sorghum plants were found to have higher fatty acid and lipid contents compared to naïve plants [43]. Fatty acids are reported to confer resistance by preventing pathogen penetration and proliferation through the strengthening of membranes and cell walls [75]. Also, they are major constituents of cutin and waxy polyester matrices, forming a protecting film that controls the fluxes of gases and water and prevents easy entry of harmful substances and pathogens into the plant [76,77]. Lastly, fatty acids are the precursors for oxylipins, including jasmonic acid and azelaic acid, which play crucial roles in the regulation of plant defense responses [75].

#### 3.2.5. Glycoalkaloids as Markers of PGPR Priming

Steroidal alkaloids (SAs) and their glycosylated forms (steroidal glycoalkaloids—SGAs) are nitrogen-containing molecules mainly found in Solanaceae vegetable crops [78]. Although these molecules confer plant resistance to a wide range of pathogens and herbivores, they are considered anti-nutritional molecules due to their toxicity to humans [79]. SAs and SGAs (Table 1) were detected in the tissues under investigation, and both tissue- and strain-dependent accumulation was observed (Figure 5). Moreover, these effects were also observed to occur as time-dependent perturbations in post-inoculation. Previous studies on the ability of SAs and SGAs to confer resistance have shown varying outcomes [79,80]. For example, α-tomatine have been shown to inhibit mycelial growth in a spectrum of fungal pathogens [81,82]. Surprisingly, the same molecule stimulated *Phytophthora infestans* mycelia growth while inhibiting zoospore germination [83]. However, studies have shown that these molecules are less effective with the removal or one or more of the sugar components [81,84,85,86]. In addition, microbes have evolved to reduce the activity of tomatidine by removing the sugar residues, and enzymes encoding glycoside hydrolases have been proposed in *P. infestans* [87]

## 4. Experimental Procedures

### 4.1. Bacterial Cultures and Growth Conditions

Four PGPR strains (*Pseudomonas fluorescens* (N04)*, Ps. koreensis* (N19)*, Paenibacillus alvei* (T22), and *Lysinibacillus sphaericus* (T19)) were obtained from the culture collection of Professor Nico Labuschagne, University of Pretoria, South Africa. These PGPR have previously been reported to successfully colonize tomato, maize, and wheat roots and enhanced the growth of these plants [88,89]. Some of the elucidated mechanisms by which these bacterial isolates enhanced plant growth include the production of siderophores, indole-acetic acid and related compounds and phosphate solubilization [88]. *Pa. alvei* T22 was also found to be an effective biocontrol agent against soil borne diseases in wheat and sorghum [43,88,90]. These strains were grown in Luria-Bertani (LB) medium at 28 °C and stocks were maintained in LB medium containing 15% (*v/v*) glycerol at −80 °C. 

### 4.2. Plant Material and PGPR Treatment

Tomato (*Solanum lycopersicum* cv. Moneymaker) seeds were sowed, germinated, and grown in potted, washed, and autoclaved playpen sand under controlled greenhouse conditions (minimum temperature 15 °C and maximum temperature 28 °C, light/dark cycle of 12 h/12 h, and light intensity of 60 μmol/m^2^/s). The plants were watered with distilled water and treated with a fertilizer mixture on a weekly basis. The latter consisted of the following: 650 mg/L CaNO_3_, 400 mg/L KNO_3_, 300 mg/L MgSO_4_, 90 mg/L *mono*-ammonium phosphate, 90 mg/L *mono*-potassium phosphate, 150 mg/L Soluptase, 20 mg/L Microples, and 40 µL/L Kep-P-Max obtained from Shiman SA (Olifantsfontein, Gauteng, South Africa). The plants were grown for eight weeks before PGPR treatment. The four bacterial strains were grown in Luria broth (LB) overnight at 28 °C and the optical density (OD_600_) was adjusted to 0.5–0.6 in 200 mL LB broth. The diluted (3 mL) culture was applied on plant roots while control plants received only the diluted LB broth with no bacteria. Bacterial growth rates and viability post-inoculation were not investigated. At 24 h and 48 h post-treatment, the leaves, stems and roots were harvested separately, frozen in liquid nitrogen, and stored at –80 °C until metabolite extraction. 

### 4.3. Aromatic Amino Acid Quantification

#### 4.3.1. Standard Curve Generation 

Stock solutions of 5 mg/mL for authentic standards of _L_-Phe, _L_-Try and _L_-Trp as well as the internal standard, prednisolone (Pred), were prepared in 50% methanol (Romil Pure Chemistry, Cambridge, UK). The stock solutions were diluted to the following concentrations: 0.004, 0.04, 0.4, 1, 3, 7, 12, and 20 ng/µL and stored at 4 °C until analyzed.

#### 4.3.2. Plant Extracts 

To extract aromatic amino acids from treated and non-treated tissue, the samples were subjected to the extraction procedure previously described [91], with minor modifications. In short, 200 mg of ground material was suspended in 1 mL ice-cold MeOH:H_2_O (50/50, *v/v*, −20 °C) in 2 mL microcentrifuge “BashingBead” lysis tubes containing ceramic microbeads (Zymo Research, Irvine, CA, USA). Pred (1 ng/µL working concentration) was used as internal standard, owing to the high cost and procurement difficulty involved with stable isotope-based internal standards [91,92]. The samples were further disrupted with a FastPrep FP120 instrument at 5 °C for 3 min at high speed. The extracts were centrifuged within the lysis tubes at 13,000× *g* and 5 °C, the supernatants transferred into clean 2 mL microcentrifuge tubes, and the pellets re-extracted with 0.5 mL ice-cold MeOH:H_2_O (50/50, *v/v*, −20 °C). The samples were combined and passed through a 0.22 µm syringe filter into 2 mL chromatography vials with slitted caps and stored at −20 °C until analysis. 

#### 4.3.3. Recovery

The recovery percentage was calculated comparing the concentration of each amino acid present in spiked/extracted (spiked with 1, 3, and 10 ng/µL of aromatic amino acids) and quality control (1, 3, and 10 ng/µL) samples. Both the spiked/extracted and quality control samples were then taken through the extraction procedure described above. 

#### 4.3.4. MRM Optimization, Selectivity/Specificity, Limit of Detection, and Quantification

Direct infusion into the MS using the electrospray ionization (ESI) source was used to optimize the MS conditions of the standards (amino acids and internal standard). The “MRM optimation method tool,” a component of the LabSolution LCMS software (Shimadzu Corporation, Kyoto, Japan), was used to optimize each transmission energy. This tool collects the product ion scan data and finds the optimum collision energy of each transition. The retention times (Rts) and the MRM transitions utilized for quantification of the aromatic amino acids and internal standard are outlined in Table S1. 

Selectivity is defined as the ability of the developed quantification method to discriminate the analytes from the various components present in the methanol extracts, thus producing well-resolved and symmetrical peaks. For methods employing both LC and MS, selectivity can be assessed by chromatographic resolution and MS/MS spectra (MRM transition fragments) [87]. Thus, the method was selective for amino acid quantification since the MRM chromatograms (Figure S1, due to the large number of figures only examples are shown and the data can be made available upon request) showed one symmetrical peak for each of the amino acids. Also, the MS/MS spectra were used to assess selectivity by comparing the standards’ spectra with that of plant samples (Figure S2; due to the large number of figures, only examples are shown, and the data can be made available upon request), with the latter being similar to the former. The limit of detection (LOD) is defined as the smallest concentration that can be distinguished from zero with a defined degree of confidence. In the present study, the LOD of the instrument was detected by diluting amino acid standards, and the values are summarized in Table S2. Furthermore, the limit of quantification (LOQ) (Table S2) is the lowest concentration of specific analytes that can be quantified with high precision and accuracy.

#### 4.3.5. Statistical Analysis

LC-MS data acquisition was carried out in triplicate and the results were expressed as mean values ± standard deviation (SD). SPSS software (IBM SPSS Statistics for Windows, Version 26. IBM Corp., Armonk, NY, USA) was used for such descriptive statistics. Here, univariate analysis of variance (ANOVA) was performed as two-tailed complete randomized blocks and used to compare the non-inoculated plants vs. PGPR inoculated plants at different time points. ANOVA was followed by the Tukey post-hoc test where differences between the means were considered significant at *p* < 0.05, indicated in graphs with an asterix (*) or a dot (•). The summarized outputs are presented in Tables S3 and S4.

### 4.4. UHPLC-QqQ-MS Analysis

Two microliters of the 50% methanol extracts were analyzed on an ‘Nexera’UHPLC system, (Shimadzu Corporation, Kyoto, Japan), coupled to a triple quadrupole mass spectrometer (QqQ-MS) (Shimadzu Corporation, Kyoto, Japan). The aqueous-methanol extracts were chromatographically separated on an ‘Ultra AQ’ C18 column (100 mm × 2.1 mm × 3 µm) (Restek Corporation, Bellefonte, PA, USA), thermostatted at 40 °C. Analytes were eluted with a binary solvent system consisting of 0.1% aqueous formic acid in water (Sigma-Aldrich, Taufkirchen, Germany) (solvent A) and 0.1% formic acid in acetonitrile (Romil Pure Chemistry, Cambridge, UK) (solvent B) at a flow rate of 0.4 mL/min. The chromatographic conditions were as follows: 15% B over 0.00–5.00 min, 15%–95% B over 5.00–11.00 min, held constant at 95% B over 11.00–14.00 min, returning from 95% to 15% B over 14.00–15.00 min. The column was washed with 15% B over 15.00–20.00 min to return to the initial conditions.

The MS conditions comprised an interface voltage set at 4.50 kV for both positive and negative ionization modes, a heat block temperature of 400 °C, an interface current of 2.75 μA, and desolvation temperature of 249 °C. Nitrogen was used as drying gas at 15.00 L/min flow rate and Argon as nebulizing gas at 1.50 L/min flow rate. The ion gauge vacuum was set at 1.5 × 10^−3^ Pa. 

### 4.5. Untargeted Metabolic Profiling

#### 4.5.1. Plant Tissue Extract Preparation for Metabolite Profiling 

The samples were ground in liquid nitrogen using a mortar and pestle, and 1 g (except for roots: 0.25 g) was dissolved in 10 mL MeOH:H_2_O (80:20, *v/v*) and sonicated for 30 min in a sonicator bath. The crude extracts were centrifuged at 4100× *g* for 20 min in a refrigerated benchtop centrifuge, after which the supernatants were evaporated to approximately 1 mL using a rotary evaporator set at 55 °C, transferred to 2 mL Eppendorf tubes, and dried to completeness in a heating block set at 55 °C. The dried residues were dissolved in 0.3 mL of MeOH:H_2_O (50:50, *v/v*), filtered through a 0.22 μm nylon filter into glass chromatography vials fitted with 500 μL inserts, and stored at −20 °C until analysis. 

#### 4.5.2. UHPLC-ESI-qTOF-MS Conditions

Three microliters of the methanol extracts were analyzed on a Waters Acquity UHPLC system coupled to a Waters photodiode array (PDA) detector and SYNAPT G1 Q-TOF mass spectrometer (Waters Corporation, Milford, MA, USA). The aqueous-methanol extracts were chromatographically separated on a Waters HSS T3 C18 column (150 mm × 2.1 mm × 1.8 µm), thermostatted at 60 °C. Analytes were eluted with a binary solvent system consisting of 0.1% aqueous formic acid and 2.5% isopropyl alcohol in water (Sigma-Aldrich, Munich, Germany) (solvent A), and 0.1% formic acid and 2.5% isopropyl alcohol in acetonitrile (Romil Pure Chemistry, Cambridge, UK) (solvent B) at a flow rate of 0.4 mL/min. The run time was 30 min and the gradient was set as follows with a flow rate of 0.4 mL/min: 2% B over 0.00–1.00 min, 2%–60% B over 1.0–22.00 min, 60%–95% B over 22.00–23.00 min, holding at 95% B over 23.00–26.00 min, and returning from 95% to 5% B over 26.00–27.00.00 min. The column was washed with 5% B over 27.00–30.00 min for column equilibration.

High definition (HD) MS analysis was performed on a Waters SYNAPT G Q-TOF MS system, and separate injections (using the same chromatographic settings and conditions) were performed for positive and negative ESI modes. The MS conditions were as listed: capillary voltage: 2.0 kV, sample cone voltage: 40 V, microchannel plate (MCP) detector voltage: 1600 V, source temperature: 120 °C and desolvation temperature: 450 °C. The cone gas flow rate was 50 L/h and the desolvation gas flow 550 L/h. The *m/z* range was 100–1500, scan time: 0.10 s, interscan delay: 0.02 s, mode: centroid. For lock mass calibration, leucine enkephalin (556.3 ng/µL) at a lock mass flow rate of 0.4 mL/min was used. The mass accuracy window was 0.5 mDa. The MassLynx^TM^ 4.1 (SCN 704, Waters Corporation, Milford, MA, USA) software was used to control the UHPLC-MS system and perform all data manipulations. Each sample of the three biological replicates was analyzed in triplicate to account for analytical variability. Pooled biological quality control (QC) samples were used to assess stability of the instrument, sample analysis reliability, reproducibility and non-linear signal correction [93,94]. To minimize instrument measurement bias and correct instrument changes, the samples were randomized, and QCs were analyzed every 10 injections. Lastly, 50% methanol samples were randomly analyzed to monitor background noise.

To assist with MS/MS annotation of both negatively and positively correlated biomarkers, the MS settings were set to perform unfragmented and five fragmenting experiments (MS^E^) simultaneously by ramping in-source collision energy from 3 eV to 30 eV [31,32,35,36,37].

### 4.6. Multivariate Data Analysis (MVDA)

The acquired UHPLC-qTOF-MS raw data sets were pre-processed using MarkerLynx XS™ software (Waters, Milford, MA, USA), and both ESI negative and ESI positive raw data were analyzed. Data normalization was done by using total ion intensities of each defined peak. Prior to calculating intensities, the software performs a patented modified Savitzky-Golay smoothing and integration. For multivariate modeling the data matrixes were created using the following parameters: retention time (Rt) range 2.5–25 min, Rt difference 0.2, mass to charge ratio (*m/z*) range 100–2000, *m/z* difference 0.05, mass tolerance 0.5, intensity threshold count 10 and noise level 3. The pre-processing parameters were adjusted (if needed) depending on visual inspection of the chromatograms. The resultant data matrix obtained from the MassLynx software were imported into SIMCA-P software, version 14.0 (Umetrics, Umeå, Sweden), and *Pareto* scaling was applied (to reduce the relative importance or masking effects of large values from abundant metabolites and partially preserve data structure) for multivariate statistical analysis. Principal component analysis, an unsupervized method for MVDA, was used to reduce the dimensionality of the data and summarize the information contained in the datasets, thus providing “smaller indices” that can easily be visualized and analyzed. Orthogonal-partial least square discriminant analysis, a supervised MVDA method, was used to extract maximum information of significant variables from the datasets. OPLS-DA allows the removal of systematic variation from the experimental data (X variables) that are not correlated to discriminant classes (Y) and Hotelling’s region, represented by the ellipse, define the 95% interval of confidence. The quality of the generated models is described by metabolic diagnostic tools: cumulative model variation in the matrix X, goodness-of-fit parameter [*R^2^X(cum)*], model variance proportion [*Y^2^X(cum)*] and total variation of the matrix X predicted by an extracted component [*Q^2^(cum)*] [95]. 

Furthermore, the OPLS-DA models were statistically validated using cross-validated analysis of variance (CV-ANOVA), where a *p* ≤ 0.05 indicates a good model [26], using the SIMCA software inbuilt seven-fold (default) cross-validation method and with a permutation test (10 permutations). Subsequent interpretation was facilitated by OPLS-DA S-plots, where significant ions with the correlation [*(p(corr)*] ≥ 0.6 and covariance of *(p1)* ≥ 0.05 were selected for further investigation and annotation.

### 4.7. Metabolite Annotation

Significant ions positively collerated to PGPR-treated and control plants were identified through the OPLS-DA analysis. UHPLC-qTOF-MS/MS-based “in-source collision-induced dissociation” (ISCID) was used as described elsewhere [31,36]. The *m/z* of each significant ion was used to extract a “single ion extracted chromatogram” (XIC, e.g., Figure 2A) and the spectral fragments (e.g., Figure 2B) produced by the different CEs were compared with previous data [34,35,59] and different spectral databases. The precursor ions of the extracted peaks were used to calculate putative empirical formulae, which were then used to search data bases such as Dictionary of Natural Products [96]. In addition, ChemSpider [97] was consulted for the compound identity search. The metabolites were annotated at a metabolite identification (MI) level-2 annotation [98]. In addition, the ions were also annotated based on accurate mass. The annotated metabolites are summarized in Table 1. 

## 5. Concluding Remarks and Perspectives

The data obtained in this study evidenced that PGPR inoculation of tomato plants leads to strain-specific and time-dependent metabolic reprogramming. These altered metabolomic states were discernible between different tissue types (roots, stems, and leaves). The non-targeted LC-MS global metabolomics profiling and multivariate analysis identified metabolites that exhibited altered concentrations due to plant root inoculation. They belonged to the following metabolite classes: amino acids, organic acids, HCA derivatives, flavonoids, fatty acids, benzoic acids, and glycoalkaloids. These signatory molecules represent possible biomarkers of PGPR priming in tomato. Quantification of aromatic amino acids showed that the strains modulated them differentially, as reflected by higher or lower contents in the various tissues. Combined, the results indicate that the differential modulation of the identified metabolites (discernible from the altered metabolic profiles in response to the four PGPR strains) are dependent on the perceived stimuli that originate from the bacteria and that the plants fine-tune their adaptive/defense responses based on the signal(s) perceived. The altered levels of these metabolites could potentially increase the performance of plants inoculated with PGPR for the agricultural industry. As such, the data provides information that can form a foundation for further investigations, elucidating the underlying mechanisms in support of PGPR priming. 

## Figures and Tables

**Table 1:** Summary of annotated metabolites that contributed to the discriminating variability in the altered/primed metabolomes as described by chemometric models. These discriminating metabolites were identified based on OPLS-DA S-plots, with a rigorous statistical validation. The reported metabolites had VIP scores ˃ 1.0 and were annotated to MSI level 2.

No.	Rt	*m/z*	Ionization	MS/MS Fragments	Compound Name	Abbreviation
1	2.56	371.058	[M − H]^−^	209, 191, 147,135	Caffeoylglucaric acid I	CGA I
2	2.56	353.075	[M − H]^−^	191, 179, 135	3-Caffeoylquinic acid	3-CQA
3	2.71	203.079	[M − H]^−^	142, 116	Tryptophan	Trp
4	2.72	371.054	[M − H]^−^	209, 191, 147,135	Caffeoylglucaric acid II	CGA II
5	3.92	385.087	[M − H]^−^	223, 208, 179	Sinapoyl glycoside	S-glyc I
6	3.93	367.157	[M − H]^−^	191,179, 173, 135	*trans*-4-Feruloylquinic acid	*t-*4-FQA
8	3.97	707.182	[M − H]^−^	353, 191, 135	5-Caffeoylquinic acid	5-CQA
9	4.08	367.157	[M − H]^−^	191, 135	*trans*-5-Feruloylquinic acid	*t-*5-FQA
10	4.16	367.157	[M − H]^−^	191, 135	*trans*-5-Feruloylquinic acid	*c-*5-FQA
11	4.3	385.106	[M − H]^−^	223, 208, 179	Sinapoyl glycoside	S-glyc II
12	4.35	353.085	[M − H]^−^	191, 135	5-Caffeoylquinic acid	5-CQA
14	4.7	355.101	[M − H]^−^	193	Feruloylglycoside I	F-glyc I
15	4.7	401.141	[M − H]^−^	293, 269, 233, 191, 161, 149, 131, 125, 101	Benzyl alcohol hexose-pentose	Be-acl hex-pent
16	4.87	385.107	[M − H]^−^	223, 208, 179	Sinapoylglycoside	S-glyc III
17	4.97	353.085	[M − H]^−^	191, 179, 173, 135	4-Caffeoylquinic acid	4-CQA
18	5.07	411.172	[M − H]^−^	321, 249, 135	Caffeoylputrescine glycoside	C-putr glyc I
19	5.15	355.110	[M − H]^−^	193	Feruloylglycoside isomer	F-glyc II
20	5.71	385.184	[M − H]^−^	223, 208, 179	Sinapoylglycoside	S-glyc IV
21	5.75	441.124 431.155	[M − H]^−^	269, 223, 161, 113, 101	Benzyl alcohol dihexose	Be-alc dihex I
22	5.77	411.184	[M − H]^−^	321, 249, 179, 135	Caffeoylputrescine glycoside	C-put glyc II
23	6.5	337.148	[M − H]^−^	191, 163, 119	5-*p*-Coumaroylquinic acid	5-*p*-CoQA
24	6.92	245.009	[M − H]^−^	203, 142, 116	Acetyl tryptophan	Acetyl Trp
25	7.42	609.142	[M − H]^−^	300	Rutin	Rutin
26	7.55	355.120	[M − H]^−^	193	Feruloylglycoside II	F-glyc II
27	7.72	449.195	[M − H]^−^	287	Eriodictyol glycoside I	Eri-glyc I
28	8.2	335.051	[M − H]^−^	179	3-Caffeoylshikimic acid	3-CSA
29	8.28	441.191 431.156	[M − H]^−^	269, 223, 161, 113, 101	Benzyl alcohol dihexose	B-alc-dihex II
30	8.3	449.164	[M − H]^−^	287	Eriodictyol glycoside II	Eir-glyc II
31	8.84	449.191	[M − H]^−^	287	Eriodictyol glycoside III	Eri-glyc III
32	9.03	949.251	[M + H]^+^	787, 479, 317	Petunidin-3-(caffeoyl) -rutinoside-5-O-glucoside	Pe-3-(C)-rut-5-O-gluc
33	9.19	1032.184	[M + H]^+^	576, 414	Dehydrotomatine	De-tom I
34	9.93	282.112	[M − H]^−^	164	Guanosine	Gua
35	10.01	1032.184	[M + H]^+^	576, 414	Dehydrotomatine	De-tom II
36	11.04	1032.185	[M + H]^+^	576, 527, 414	Dehydrotomatine	De-tom III
37	11.19	1034.546	[M + H]^+^	578, 528, 416	α-Tomatine I	α-Tom I
38	11.48	1034.547	[M + H]^+^	578, 528, 416	α-Tomatine II	α-Tom II
39	11.71	447.221	[M − H]^−^	315	Isorhamnetin pentoside	Iso-pent
40	11.75	1034.188	[M + H]^+^	578, 528, 416	α-Tomatine II	α-Tom III
41	11.88	474.181	[M − H]^−^	312, 178	Feruloyltyramine glycoside	F-tyr glyc I
42	12.59	597.239	[M − H]^−^	273	Phloritin di-glycoside	Ph-diglyc I
43	13.35	597.239	[M − H]^−^	273	Phloritin dihexoside	Ph-diglyc II
44	17.99	593.281	[M − H]^−^	285	Kaempferol 3-rutinoside	Ka-3-rut
45	20.66	474.181	[M − H]^−^	312, 178	Feruloyltyramine glycoside	F-tyr glyc II
46	20.95	474.181	[M − H]^−^	312, 178	Feruloyltyramine glycoside	F-tyr glyc III
47	21.13	474.181	[M − H]^−^	312, 178	Feruloyltyramine glycoside	F-tyr glyc IV
48	22.01	546.194	[M − H]^−^	342, 178	Feruloyl-3-methoxytyramine conjugate	F-3-me-tyr glyc I
49	22.23	546.194	[M − H]^−^	342, 178	Feruloyl-3-methoxytyramine conjugate	F-3-me-tyr glyc II

## Data Availability

The study design information, LC-MS raw data, analyses and data processing information, and the meta-data were deposited to the EMBL-EBI metabolomics repository MetaboLights50, https://www.ebi.ac.uk/metabolights/MTBLS1176.

## Supplementary Materials

The following are available online at https://www.mdpi.com/2218-1989/10/5/210/s1, Figure S1: UHPLC–MRM-MS chromatograms of the aromatic amino acids in extracts from tomato root tissue inoculated with *Pseudomonas fluorescens* N04. Shown in (A) phenylalanine, (B) tryptophan, and (C) tyrosine.; Figure S2: UHPLC–MRM-MS spectra of the aromatic amino acid in extracts from tomato root tissue inoculated with Pseudomonas fluorescens N04. Shown in (A) phenylalanine, (B) tryptophan, and (C) tyrosine.; Figure S3: The PCA score and loadings plots of leaf extracts from tomato plants treated with *Pseudomonas fluorescens* N04.; Figure S4: The PCA score and loadings plots of leaf extracts from tomato plants treated with *Pseudomonas fluorescens* N19.; Figure S5: The PCA score and loadings plots of leaf extracts from tomato plants treated with *Lysinibacillus sphaericus* T19.; Figure S6: The PCA score and loadings plots of leaf extracts from tomato plants treated with *Paenibacillus alvei* T22.; Figure S7: The PCA score and loadings plots of stem extracts from tomato plants treated with *Pseudomonas koreensis* N19.; Figure S8: The PCA score and loadings plots of stem extracts from tomato plants treated with L*ysinibacillus sphaericus* T19.; Figure S9: The PCA score and loadings plots of stem extracts from tomato plants treated with *Paenibacillus alvei* T22.; Figure S10: The PCA score and loadings plots of root extracts from tomato plants treated with *Pseudomonas fluorescens* N04.; Figure S11: The PCA score and loadings plots of root extracts from tomato plants treated with *Pseudomonas koreensis* N19.; Figure S12: The PCA score and loadings plots of root extracts from tomato plants treated with *Lysinibacillus sphaericus* T19.; Figure S13: The PCA score and loadings plots of root extracts from tomato plants treated with *Paenibacillus alvei* T22.; Figure S14: Identification of discriminatory biomarkers of *Pseudomonas fluorescens* N04-treated leaf samples using OPLS-DA modeling.; Figure S15: Identification of discriminatory biomarkers of *Pseudomonas koreensis* N19-treated leaf samples using OPLS-DA modeling.; Figure S16: Identification of discriminatory biomarkers of *Lysinibacillus sphaericus* T19-treated leaf samples using OPLS-DA modeling.; Figure S17: Identification of discriminatory biomarkers of *Paenibacillus alvei* T22-treated leaf samples using OPLS-DA modeling.; Figure S18: Identification of discriminatory biomarkers of *Pseudomonas koreensis* N19-treated stem samples using OPLS-DA modeling.; Figure S19: Identification of discriminatory biomarkers of *Lysinibacillus sphaericus* T19-treated stem samples using OPLS-DA modeling.; Figure S20: Identification of discriminatory biomarkers of *Paenibacillus alvei* T22-treated stem samples using OPLS-DA modeling.; Figure S21: Identification of discriminatory biomarkers of *Pseudomonas fluorescens* N04-treated root samples using OPLS-DA modeling.; Figure S22: Identification of discriminatory biomarkers of *Pseudomonas koreensis* N19-treated root samples using OPLS-DA modeling.; Figure S23: Identification of discriminatory biomarkers of *Lysinibacillus sphaericus* T19-treated root samples using OPLS-DA modeling.; Figure S24: Identification of discriminatory biomarkers of *Paenibacillus alvei* T22-treated root samples using OPLS-DA modeling.; Figure S25: Schematic overview of branch pathways of the phenylpropanoid pathway leading to the synthesis of benzoic acids, hydroxycinnamic acid derivatives and flavonoids.; Table S1: Annotation of individual aromatic amino acids by retention time, *m/z* values and identification by MS/MS fragmentation patterns.; Table S2: Parameters of the calibration curve for each aromatic amino acid including curve range, correlation coefficient, limit of detection (LOD) and limit of quantification (LOQ)., Table S3: One-way ANOVA comparing mean values of quantified aromatic amino acids (Phe, Tyr and Trp) is roots, stems and leaves of PGPR inoculated plants.; Table S4: Post-hoc statistical tests comparing mean values of the distribution of the aromatic amino acids (Phe, Tyr and Trp) is roots, stems. and leaves of PGPR inoculated plants.
