# Supplementary material for: Metabolic Profiling of PGPR-Treated Tomato Plants Reveal Priming-Related Adaptations of Secondary Metabolites and Aromatic Amino Acids
Source: Metabolites. 2020 May 20;10(5):210. doi: 10.3390/metabo10050210 (PMC7281251; doi:10.3390/metabo10050210)
Supplement: Supplementary file 1 [file metabolites-10-00210-s001.pdf]

metabolites

MDPI

Supplementary file

Research article

Metabolic Profiling of PGPR-Treated Tomato Plants Reveal Priming-Related Adaptations of Secondary Metabolites and Aromatic Amino Acids

Msizi I. Mhlongo[1], Lizelle A. Piater[1], Paul A. Steenkamp[1] Nico Labuschagne[2] and Ian A. Dubery[1]*

Table S1. Annotation of individual aromatic amino acids by retention time, *m/z* values and identification by MS/MS fragmentation patterns. LC-ESI-MS/MS analyses were performed on a UHPLC system coupled to a triple quadrupole mass spectrometer operating in positive ion mode.

No.	Analyte	Pseudo-molecular ions	Rt (min)	*m/z*	Fragment ions (*m/z*) observed in MRM mode and relative intensity (%)	Quadrupole 1 (Q1), V	Collision energy (CE), V	Quadrupole 3 (Q3), V	Dwell time (ms)
**1**	Pred	$[M+H]^+$	8.30	361.00	361.00 > 343.25	-20.00	-10.00	-20.00	65.6
					361.00 > 325.20	-20.00	-11.00	-21.00	
					361.00 > 147.15	-19.00	-22.00	-20.00	
**2**	Phe	$[M+H]^+$	1.11	166.00	166.00 > 120.20	-12.00	-15.00	-21.00	65.6
					166.00 > 103.15	-12.00	-28.00	-18.00	
					166.00 > 77.15	-12.00	-40.00	-29.00	
**3**	Trp	$[M+H]^+$	1.43	205.05	205.05 > 188.20	-10.00	-12.00	-18.00	65.6
					205.05 > 146.15	-10.00	-19.00	-14.00	
					205.05 > 118.20	-10.00	-26.00	-11.00	
**4**	Tyr	$[M+H]^+$	0.80	182.05	182.05 > 91.20	-13.00	-30.00	-17.00	65.6
					182.05 > 136.20	-13.00	-16.00	-13.00	
					182.05 > 165.15	-10.00	-13.00	16.00	

metabolites

MDPI

Table S2. Parameters of the calibration curve for each aromatic amino acid including curve range, correlation coefficient, limit of detection (LOD) and limit of quantification (LOQ).

Compound	Range (ng/µL FW)	Curve	R^2	LOD (ng/µL)	LOQ (ng/µL)
Pred	0.0001 – 20	Y = 1.59^{6}X + 717 037	99.54	> 0.001	0.001
Phe	0.0001 – 20	Y = 7.98^{6}X + 1.16^{6}	99.50	> 0.0001	0.0001
Trp	0.0001 – 20	Y = 8.21^{6}X + 4.52^{6}	99.10	> 0.0001	0.0001
Tyr	0.0001 – 20	Y = 520 669X + 411 370	97.20	> 0.0001	0.0001

Table S3. One-way ANOVA comparing mean values of quantified aromatic amino acids (Phe, Tyr and Trp) in roots (R), stems (S) and leaves (L) of PGPR-inoculated plants.

Compound	N04 *p*-value	N19 *p*-value	T19 *p*-value	T22 *p*-value
**Roots**				
Phe	0.000	0.001	0.000	0.000
Trp	0.000	0.010	0.000	0.000
Tyr	0.000	0.000	0.000	0.000
**Stems**				
Phe	0.000	0.000	0.000	0.000
Trp	0.000	0.000	0.000	0.000
Tyr	0.000	0.000	0.000	0.000
**Leaves**				
Phe	0.000	0.000	0.000	0.000
Trp	0.000	0.000	0.000	0.000
Tyr	0.000	0.000	0.000	0.000

Table S4. Post-hoc statistical tests comparing mean values of the distribution of the aromatic amino acids (Phe, Tyr and Trp) is roots, stems and leaves of PGPR-inoculated plants.

Compound Name	Treatment	Treatment	Significance (*p*).
**Roots**			
Phe	NT R Day 1	NT R Day 2	1.000
		N04 R Day 1	0.000
		N04 R Day 2	0.000
		N19 R Day 1	0.000
		N19 R Day 2	0.659
		T19 R Day 1	0.000
		T19 R Day 2	0.000
		T22 R Day 1	0.000
		T22 R Day 2	0.000
	NT R Day 2	NT R Day 1	1.000
		N04 R Day 1	0.000

		N04 R Day 2	0.000
		N19 R Day 1	0.000
		N19 R Day 2	0.739
		T19 R Day 1	0.000
		T19 R Day 2	0.000
		T22 R Day 1	0.000
		T22 R Day 2	0.000
Trp	NT R Day 1	NT R Day 2	0.883
		N04 R Day 1	0.000
		N04 R Day 2	0.000
		N19 R Day 1	0.000
		N19 R Day 2	0.000
		T19 R Day 1	0.000
		T19 R Day 2	0.000
		T22 R Day 1	0.000
		T22 R Day 2	0.016
	NT R Day 2	NT R Day 1	0.883
		N04 R Day 1	0.000
		N04 R Day 2	0.000
		N19 R Day 1	0.000
		N19 R Day 2	0.000
		T19 R Day 1	0.000
		T19 R Day 2	0.000
		T22 R Day 1	0.000
		T22 R Day 2	0.000
Tyr	NT R Day 1	NT R Day 2	0.665
		N04 R Day 1	0.005
		N04 R Day 2	0.254
		N19 R Day 1	0.062
		N19 R Day 2	0.999
		T19 R Day 1	0.286
		T19 R Day 2	0.001
		T22 R Day 1	0.000
		T22 R Day 2	0.000
	NT R Day 2	NT R Day 1	0.665
		N04 R Day 1	0.000
		N04 R Day 2	0.001
		N19 R Day 1	0.000
		N19 R Day 2	0.229

		T19 R Day 1	0.001
		T19 R Day 2	0.000
		T22 R Day 1	0.000
		T22 R Day 2	0.000
		Stems	
Phe	NT S Day 1	NT S Day 2	1.000
		N04 S Day 1	0.000
		N04 S Day 2	0.000
		N19 R Day 1	0.000
		N19 R Day 2	0.000
		T19 S Day 1	0.000
		T19 S Day 2	0.000
		T22 S Day 1	0.000
		T22 S Day 2	0.000
	NT S Day 2	NT S Day 1	1.000
		N04 S Day 1	0.000
		N04 S Day 2	0.000
		N19 R Day 1	0.000
		N19 R Day 2	0.000
		T19 S Day 1	0.000
		T19 S Day 2	0.000
		T22 S Day 1	0.000
		T22 S Day 2	0.000
Trp	NT S Day 1	NT S Day 2	1.000
		N04 S Day 1	0.000
		N04 S Day 2	0.000
		N19 S Day 1	0.000
		N19 S Day 2	0.000
		T19 S Day 1	0.000
		T19 S Day 2	0.000
		T22 S Day 1	0.000
		T22 S Day 2	0.000
	NT S Day 2	NT S Day 1	1.000
		N04 S Day 1	0.000
		N04 S Day 2	0.000
		N19 S Day 1	0.000
		N19 S Day 2	0.000
		T19 S Day 1	0.000
		T19 S Day 2	0.000

		T22 S Day 1	0.000
		T22 S Day 2	0.000
Tyr	NT S Day 1	NT S Day 2	1.000
		N04 S Day 1	0.005
		N04 S Day 2	0.000
		N19 S Day 1	0.001
		N19 S Day 2	0.000
		T19 S Day 1	0.000
		T19 S Day 2	0.000
		T22 S Day 1	0.328
		T22 S Day 2	0.000
	NT S Day 2	NT S Day 1	1.000
		N04 S Day 1	0.039
		N04 S Day 2	0.000
		N19 S Day 1	0.007
		N19 S Day 2	0.000
		T19 S Day 1	0.000
		T19 S Day 2	0.000
		T22 S Day 1	0.749
		T22 S Day 2	0.000
**Leaves**			
Phe	NT L Day 1	NT L Day 2	1.000
		N04 L Day 1	0.578
		N04 L Day 2	0.000
		N19 L Day 1	0.000
		N19 L Day 2	0.000
		T19 L Day 1	0.000
		T19 L Day 2	0.000
		T22 L Day 1	1.000
		T22 L Day 2	0.000
	NT L Day 2	NT L Day 1	1.000
		N04 L Day 1	0.710
		N04 L Day 2	0.000
		N19 L Day 1	0.000
		N19 L Day 2	0.000
		T19 L Day 1	0.000
		T19 L Day 2	0.000
		T22 L Day 1	1.000
		T22 L Day 2	0.000

metabolites

MDPI

Trp	NT L Day 1	NT L Day 2	1.000
		N04 L Day 1	0.001
		N04 L Day 2	0.000
		N19 L Day 1	0.000
		N19 L Day 2	0.000
		T19 L Day 1	0.000
		T19 L Day 2	0.000
		T22 L Day 1	0.008
		T122L Day 2	0.000
	NT L Day 2	NT L Day 1	1.000
		N04 L Day 1	0.000
		N04 L Day 2	0.000
		N19 L Day 1	0.000
		N19 L Day 2	0.000
		T19 L Day 1	0.000
		T19 L Day 2	0.000
		T22 L Day 1	0.002
		T22 L Day 2	0.000
Tyr	NT L Day 1	NT L Day 2	0.982
		N04 L Day 1	0.297
		N04 L Day 2	0.000
		N19 L Day 1	0.000
		N19 L Day 2	0.000
		T19 L Day 1	0.000
		T19 L Day 2	0.000
		T22 L Day 1	0.934
		T22 L Day 2	0.000
	NT L Day 2	NT L Day 1	0.982
		N04 L Day 1	0.936
		N04 L Day 2	0.000
		N19 L Day 1	0.000
		N19 L Day 2	0.000
		T19 L Day 1	0.000
		T19 L Day 2	0.000
		T22 L Day 1	1.000
		T22 L Day 2	0.000

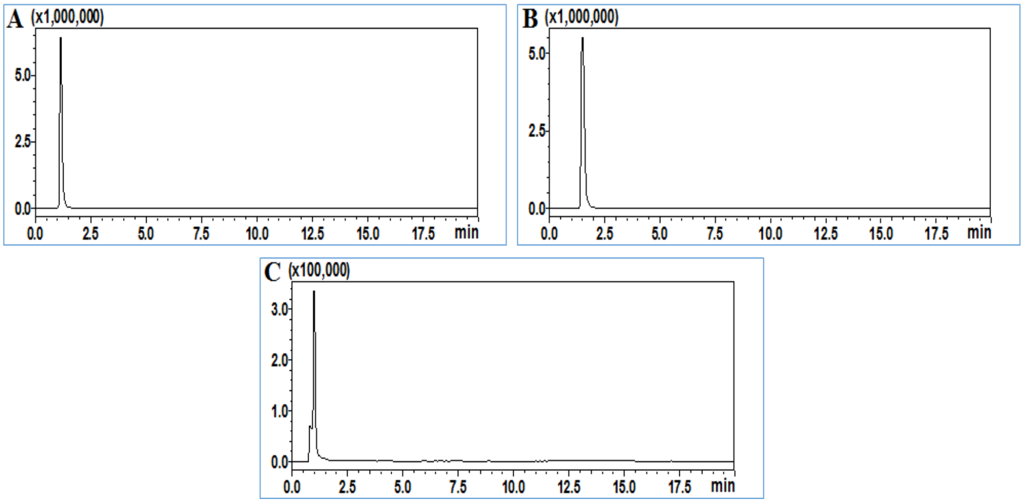

Figure S1. UHPLC–MRM-MS chromatograms of the aromatic amino acids in extracts from tomato root tissue inoculated with *Pseudomonas fluorescens* N04. Shown in (**A**): phenylalanine, (**B**): tryptophan and (**C**): tyrosine.

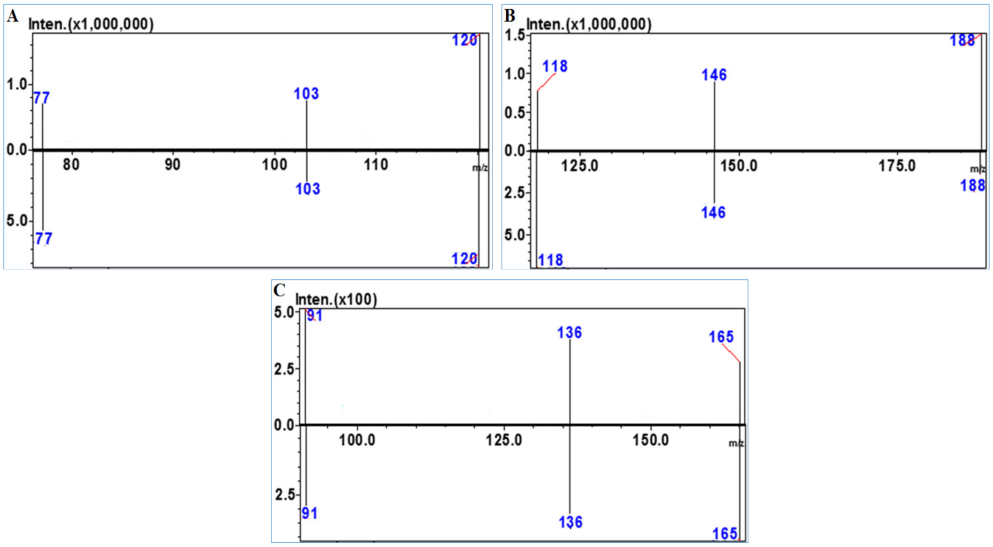

Figure S2. UHPLC–MRM-MS spectra of the aromatic amino acid in extracts from tomato root tissue inoculated with *Pseudomonas fluorescens* N04. Shown in (**A**): phenylalanine, (**B**): tryptophan and (**C**): tyrosine.

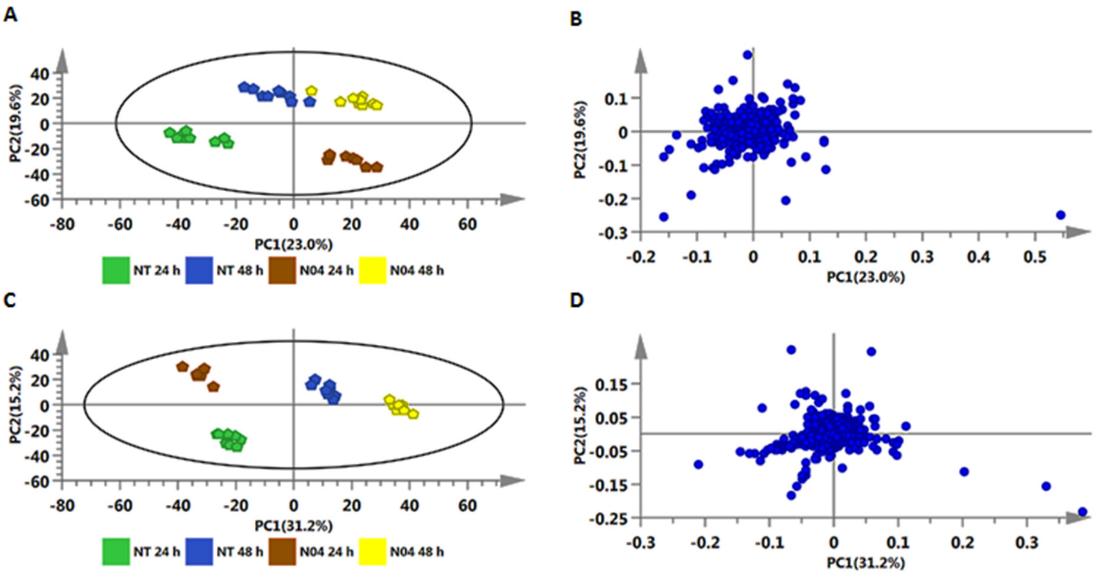

Figure S3. The PCA score - and loadings plots of leaf extracts from tomato plants treated with *Pseudomonas fluorescens* N04. The PCA scores scatter plots (**A** and **C**) show clear separation and grouping of control and treated samples, respectively. The PCA loading plots (**B** and **D**) show ions contributing to the clustering. The X-axis and Y-axis describe the first and second PCs, respectively. (**A** and **B**) = ESI negative data and (**C** and **D**) = ESI positive data.

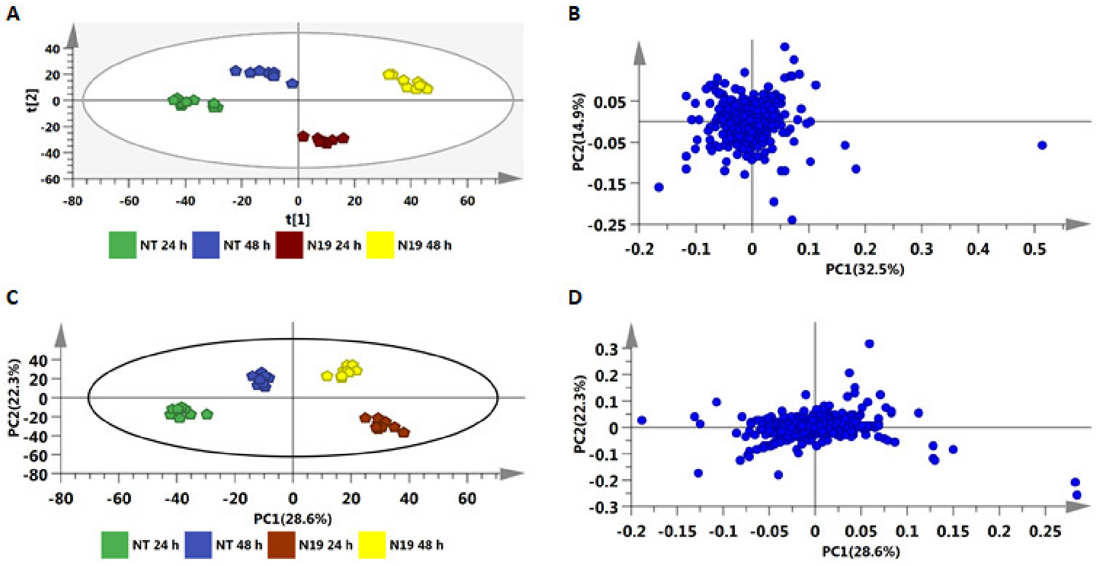

Figure S4. The PCA score - and loadings plots of leaf extracts from tomato plants treated with *Pseudomonas fluorescens* N19. The PCA scores scatter plots (**A** and **C**) show clear separation and grouping of control and treated samples, respectively. The PCA loading plots (**B** and **D**) show ions contributing to the clustering. The X-axis and Y-axis describe the first and second PCs, respectively. (**A** and **B**) = ESI negative data and (**C** and **D**) = ESI positive data.

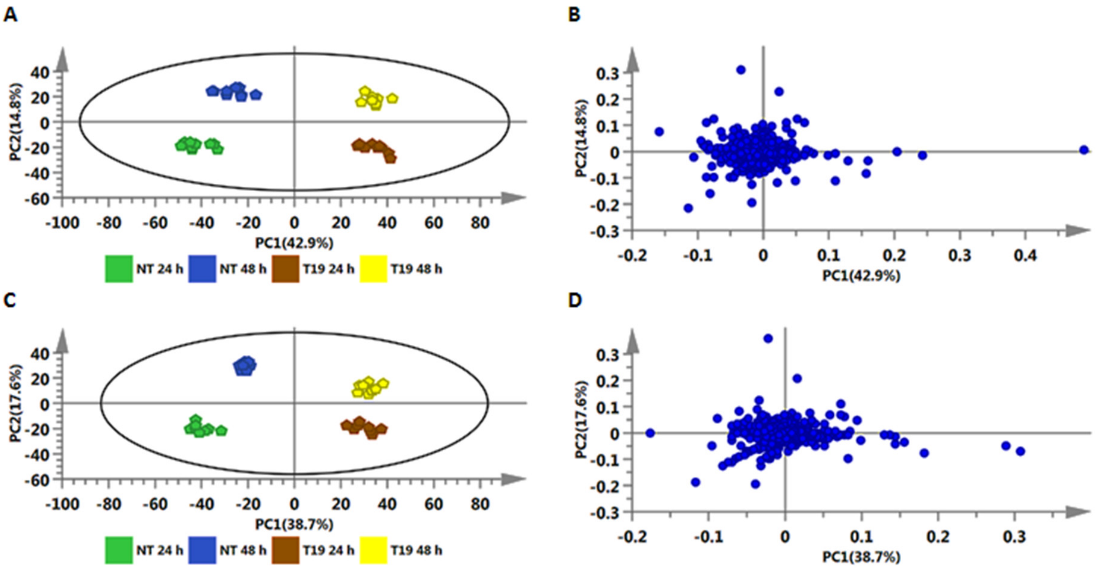

Figure S5. The PCA score - and loadings plots of leaf extracts from tomato plants treated with *Lysinibacillus sphaericus* T19. The PCA scores scatter plots (**A** and **C**) show clear separation and grouping of control and treated samples, respectively. The PCA loading plots (**B** and **D**) show ions contributing to the clustering. The X-axis and Y-axis describe the first and second PCs, respectively. (**A** and **B**) = ESI negative data and (**C** and **D**) = ESI positive data.

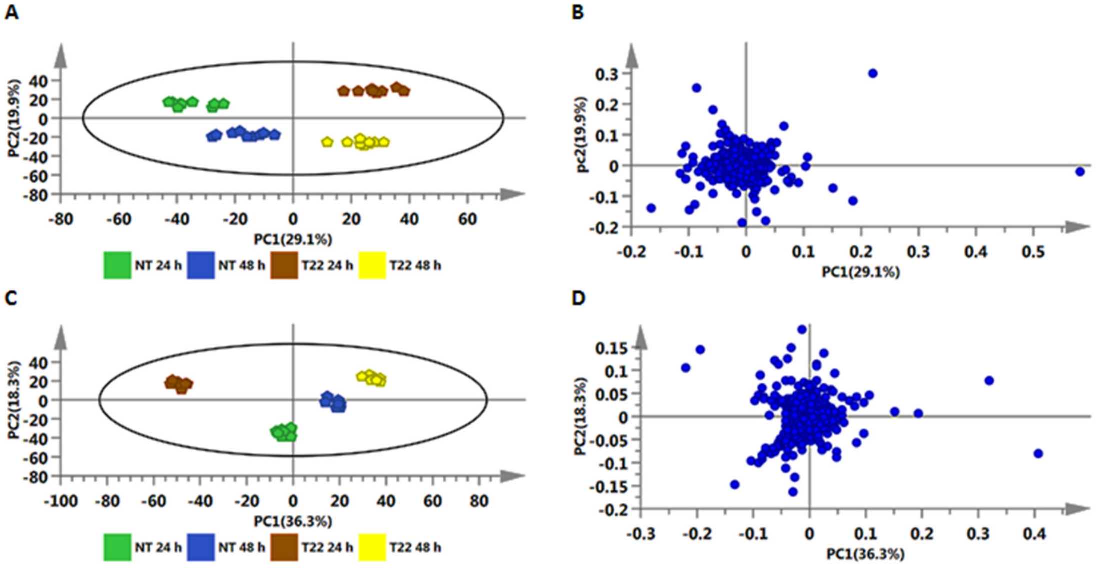

Figure S6. The PCA score - and loadings plots of leaf extracts from tomato plants treated with *Paenibacillus alvei* T22. The PCA scores scatter plots (**A** and **C**) show clear separation and grouping of control and treated samples, respectively. The PCA loading plots (**B** and D) show ions contributing to the clustering. The X-axis and Y-axis describe the first and second PCs, respectively. (**A** and **B**) = ESI negative data and (**C** and **D**) = ESI positive data.

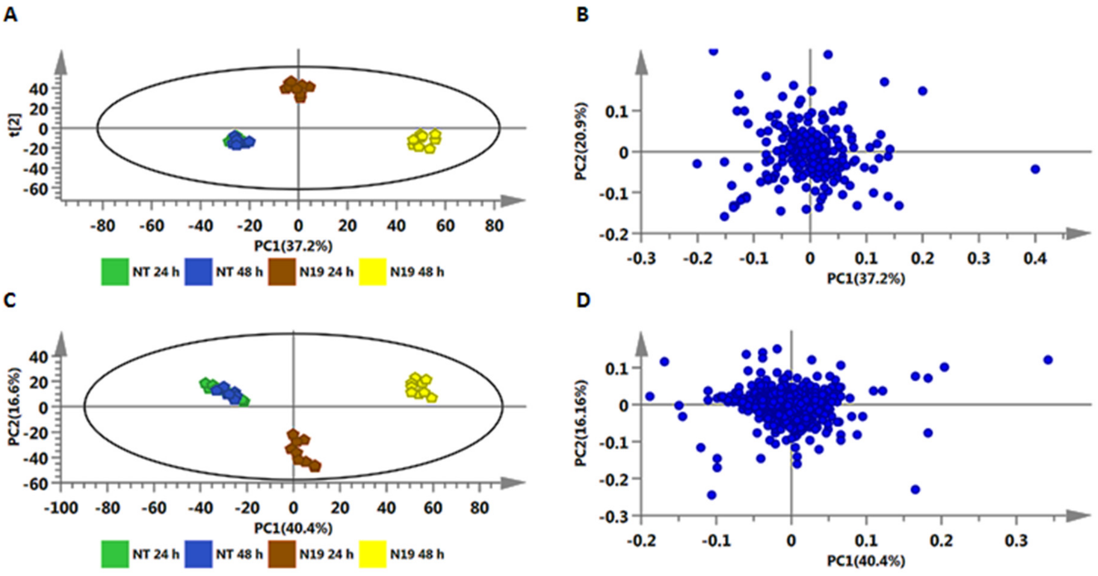

Figure S7. The PCA score - and loadings plots of stem extracts from tomato plants treated with *Pseudomonas koreensis* N19. The PCA scores scatter plots (**A** and **C**) show clear separation and grouping of control and treated samples, respectively. The PCA loading plots (**B** and **C**) show ions contributing to the clustering. The X-axis and Y-axis describe the first and second PCs, respectively. (**A** and **B**) = ESI negative data and (**C** and **D**) = ESI positive data.

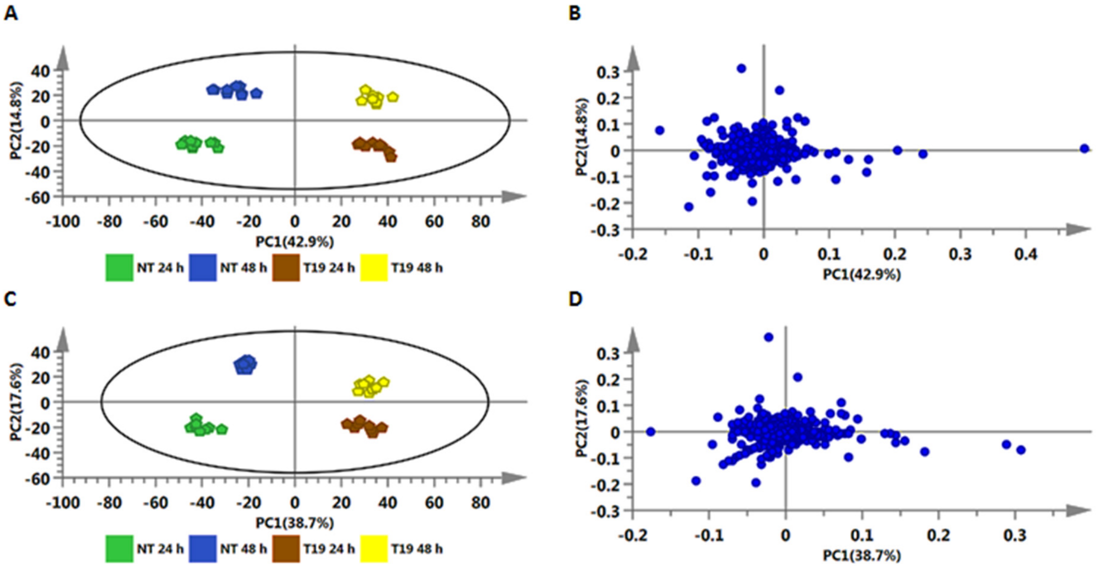

Figure S8. The PCA score - and loadings plots of stem extracts from tomato plants treated with *Lysinibacillus sphaericus* T19. The PCA scores scatter plots (**A** and **C**) show clear separation and grouping of control and treated samples, respectively. The PCA loading plots (**B** and **D**) show ions contributing to the clustering. The X-axis and Y-axis describe the first and second PCs, respectively. (**A** and **B**) = ESI negative data and (**C** and **D**) = ESI positive data.

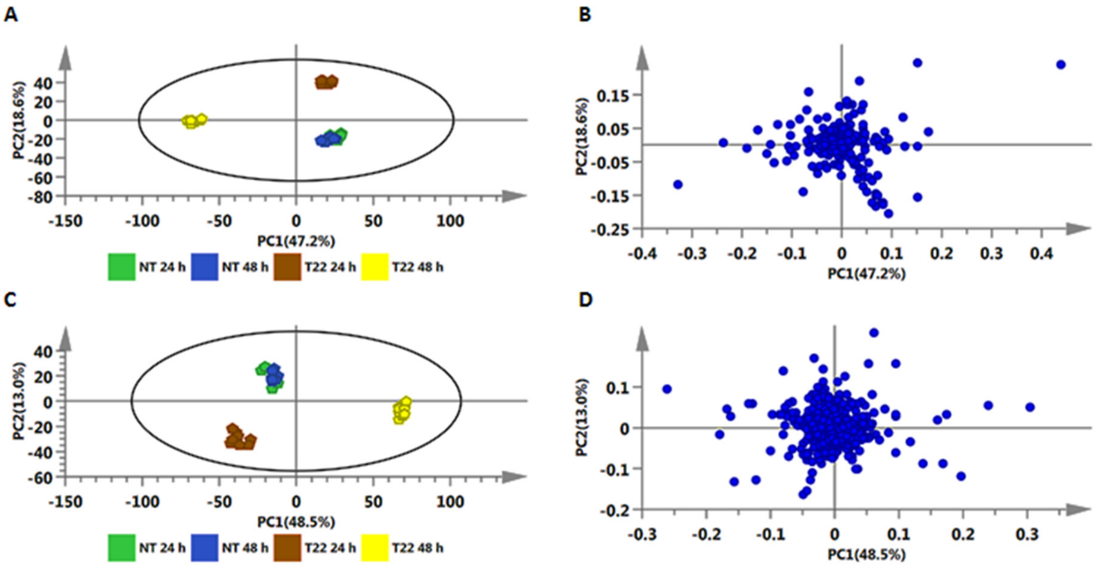

Figure S9. The PCA score - and loadings plots of stem extracts from tomato plants treated with *Paenibacillus alvei* T22. The PCA scores scatter plots (**A** and **C**) show clear separation and grouping of control and treated samples, respectively. The PCA loading plots (**B** and **D**) show ions contributing to the clustering. The X-axis and Y-axis describe the first and second PCs, respectively. (**A** and **B**) = ESI negative data and (**C** and **D**) = ESI positive data.

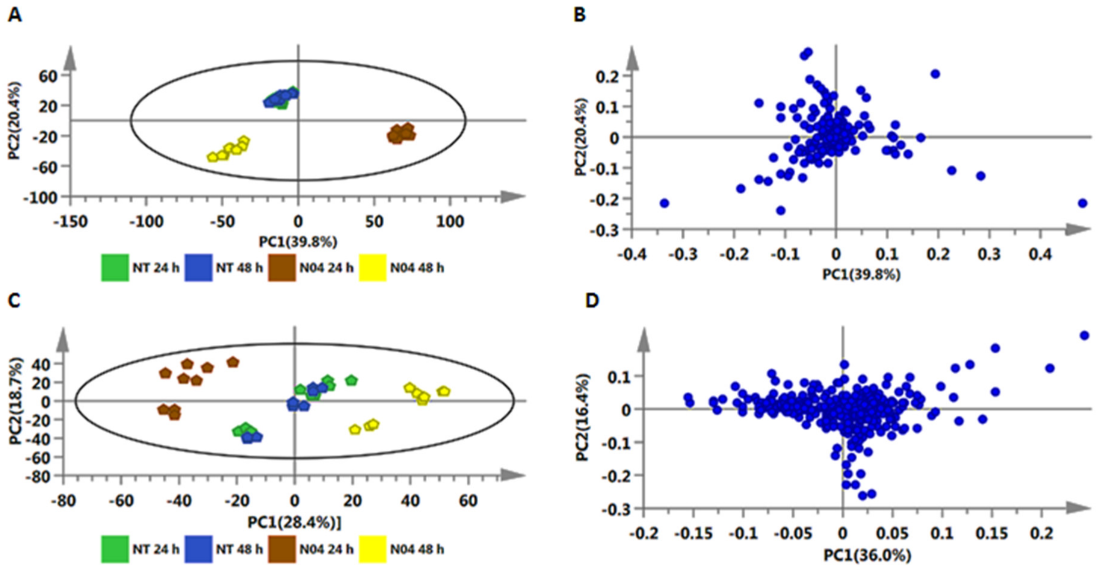

Figure S10. The PCA score - and loadings plots of root extracts from tomato plants treated with *Pseudomonas fluorescens* N04. The PCA scores scatter plots (**A** and **C**) show clear separation and grouping of control and treated samples, respectively. The PCA loading plots (**B** and **D**) show ions contributing to the clustering. The X-axis and Y-axis describe the first and second PCs, respectively. (**A** and **B**) = ESI negative data and (**C** and **D**) = ESI positive data.

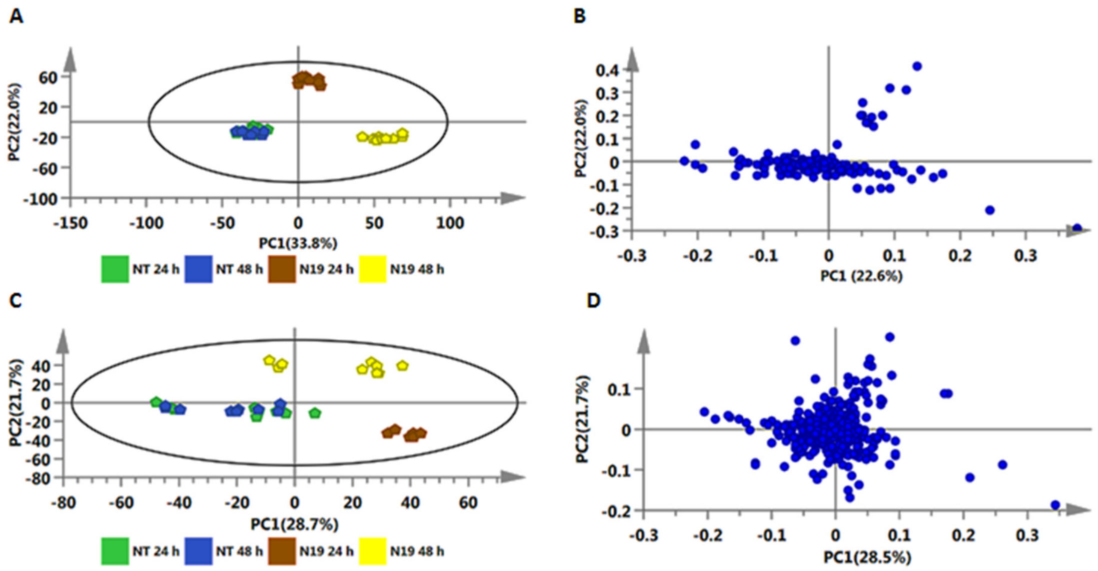

Figure S11. The PCA score - and loadings plots of root extracts from tomato plants treated with *Pseudomonas koreensis* N19. The PCA scores scatter plots (**A** and **C**) show clear separation and grouping of control and treated samples, respectively. The PCA loading plots (**B** and **D**) show ions contributing to the clustering. The X-axis and Y-axis describe the first and second PCs, respectively. (**A** and **B**) = ESI negative data and (**C** and **D**) = ESI positive data.

.

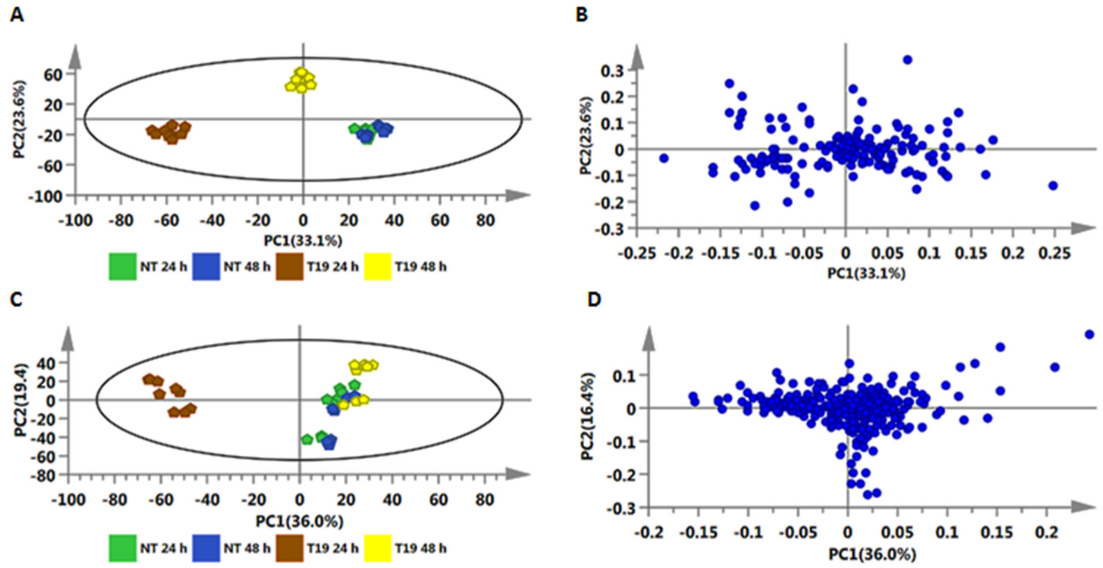

Figure S12. The PCA score - and loadings plots of root extracts from tomato plants treated with *Lysinibacillus sphaericus* T19. The PCA scores scatter plots (**A** and **C**) show clear separation and grouping of control and treated samples, respectively. The PCA loading plots (**B** and **D**) show ions contributing to the clustering. The X-axis and Y-axis describe the first and second PCs, respectively. (**A** and **B**) = ESI negative data and (**C** and **D**) = ESI positive data.

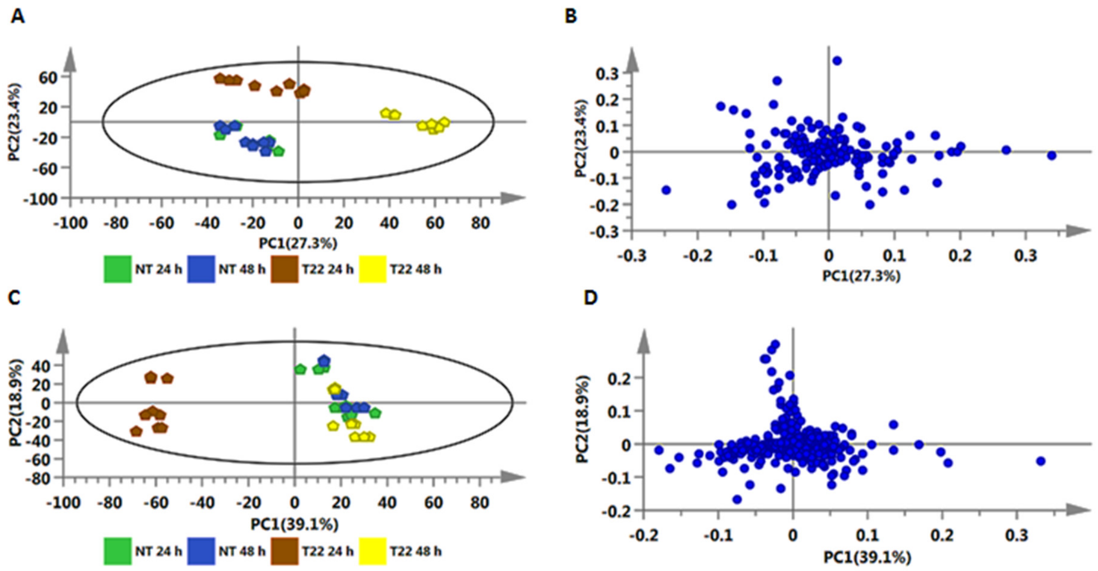

Figure S13. The PCA score - and loadings plots of root extracts from tomato plants treated with *Paenibacillus alvei* T22. The PCA scores scatter plots (**A** and **C**) show clear separation and grouping of control and treated samples, respectively. The PCA loading plots (**B** and **D**) show ions contributing to the clustering. The X-axis and Y-axis describe the first and second PCs, respectively. (**A** and **B**) = ESI negative data and (**C** and **D**) = ESI positive data.

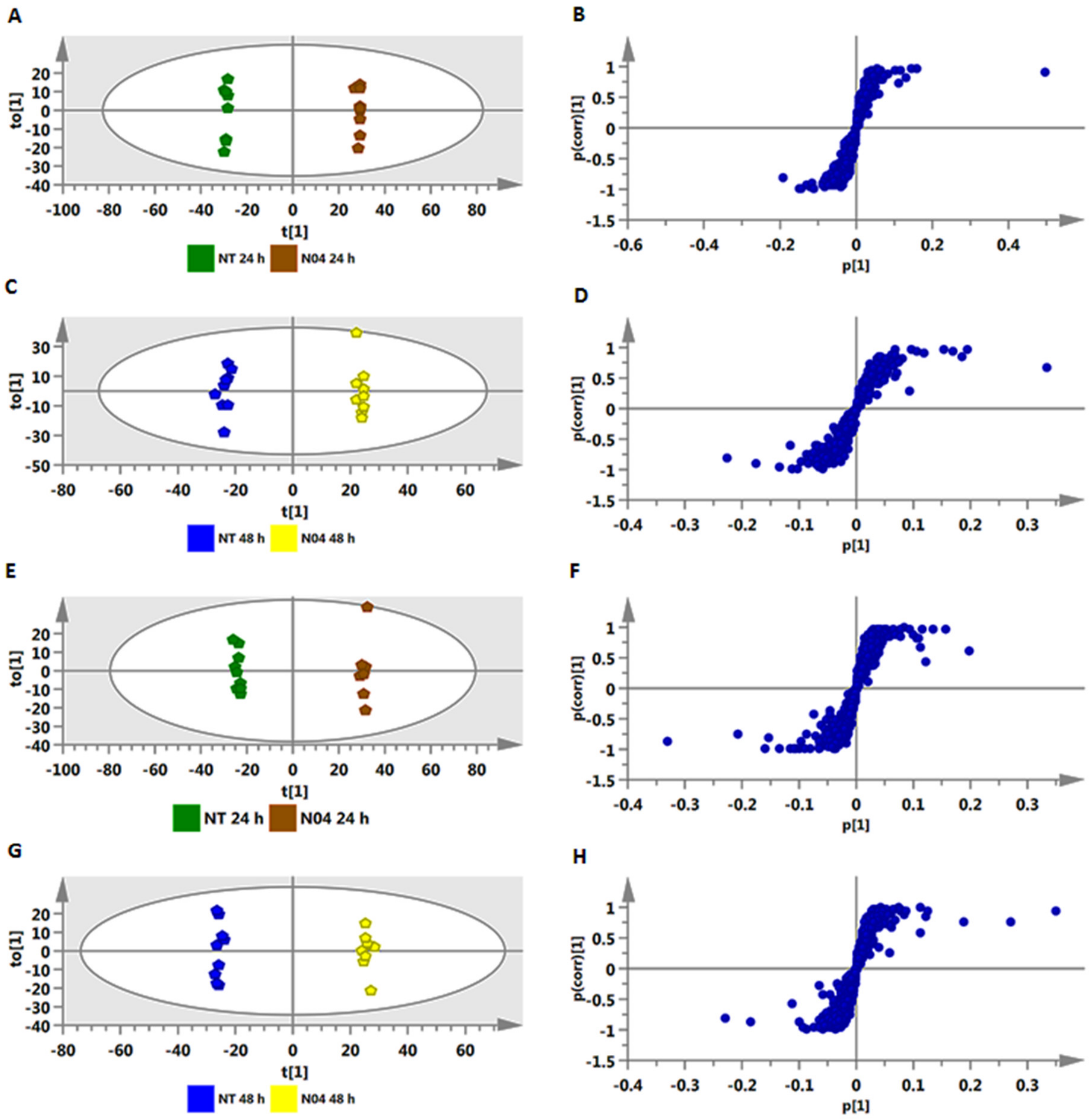

Figure S14. Identification of discriminatory biomarkers of *Pseudomonas fluorescens* N04-treated leaf samples using OPLS-DA modeling. The OPLS-DA score plots (**A, C, E** and **G**) and the corresponding S-plots (**B, D, F** and **H**) show the different clustering of treated and non-treated samples. The ellipse represents Hotelling's T^2 with 95% confidence. Model validation by CV-ANOVA showed high model significance with a *p*-value < 0.05. (**A** – **D**) = ESI negative data and (**E** – **H**) = ESI positive data.

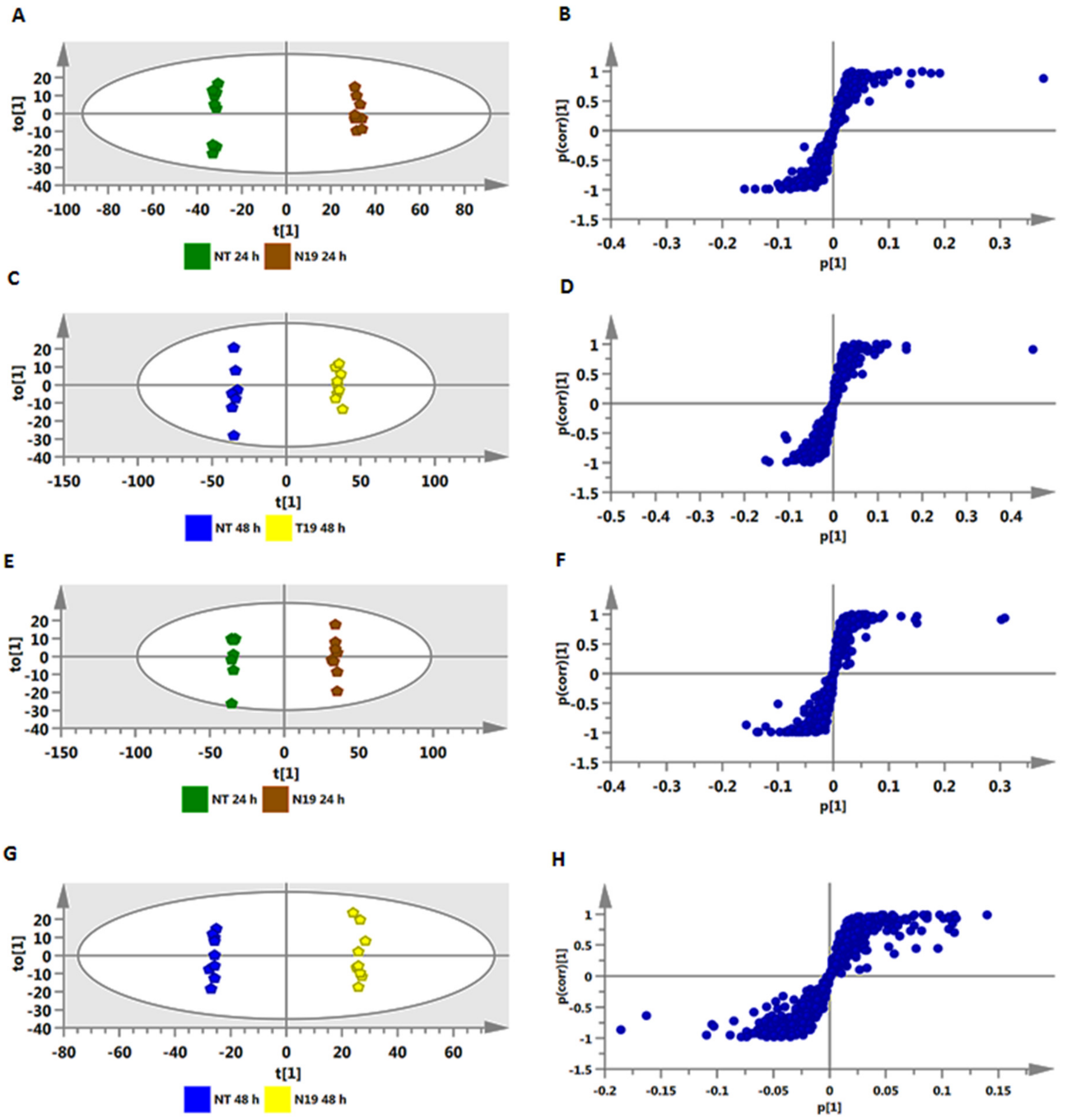

Figure S15. Identification of discriminatory biomarkers of *Pseudomonas koreensis* N19-treated leaf samples using OPLS-DA modeling. The OPLS-DA score plots (**A, C, E** and **G**) and the corresponding S-plots (**B, D, F** and **H**) show the different clustering of treated and non-treated samples. The ellipse represents Hotelling's T^2 with 95% confidence. Model validation by CV-ANOVA showed high model significance with a p-value < 0.05. (**A – D**) = ESI negative data and (**E – H**) = ESI positive data.

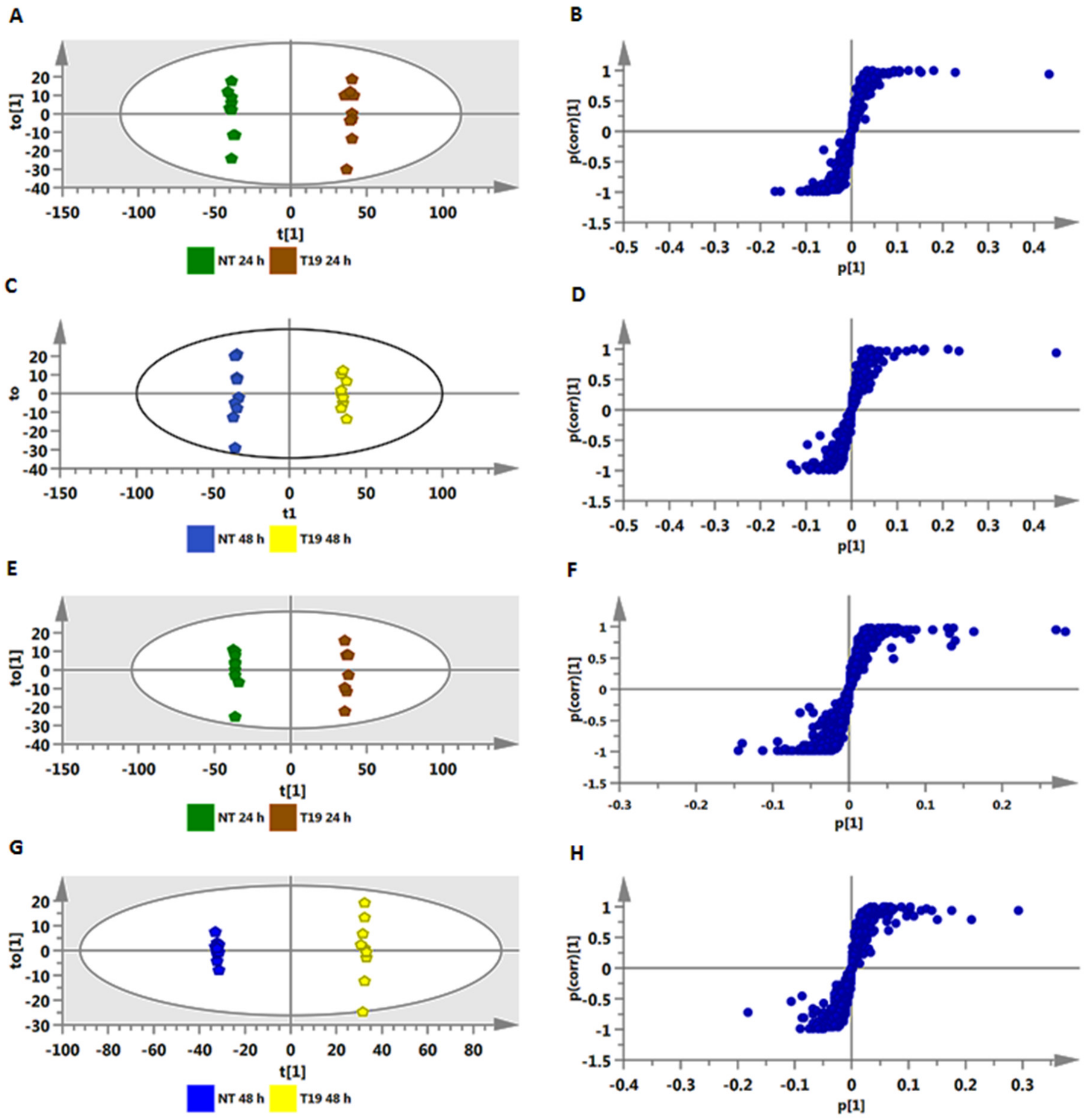

Figure S16. Identification of discriminatory biomarkers of *Lysinibacillus sphaericus* T19-treated leaf samples using OPLS-DA modeling. The OPLS-DA score plots (**A, C, E** and **G**) and the corresponding S-plots (**B, D, F** and **H**) show the different clustering of treated and non-treated samples. The ellipse represents Hotelling's T^2 with 95% confidence. Model validation by CV-ANOVA showed high model significance with a *p*-value < 0.05. (**A** – **D**) = ESI negative data and (**E** – **H**) = ESI positive data.

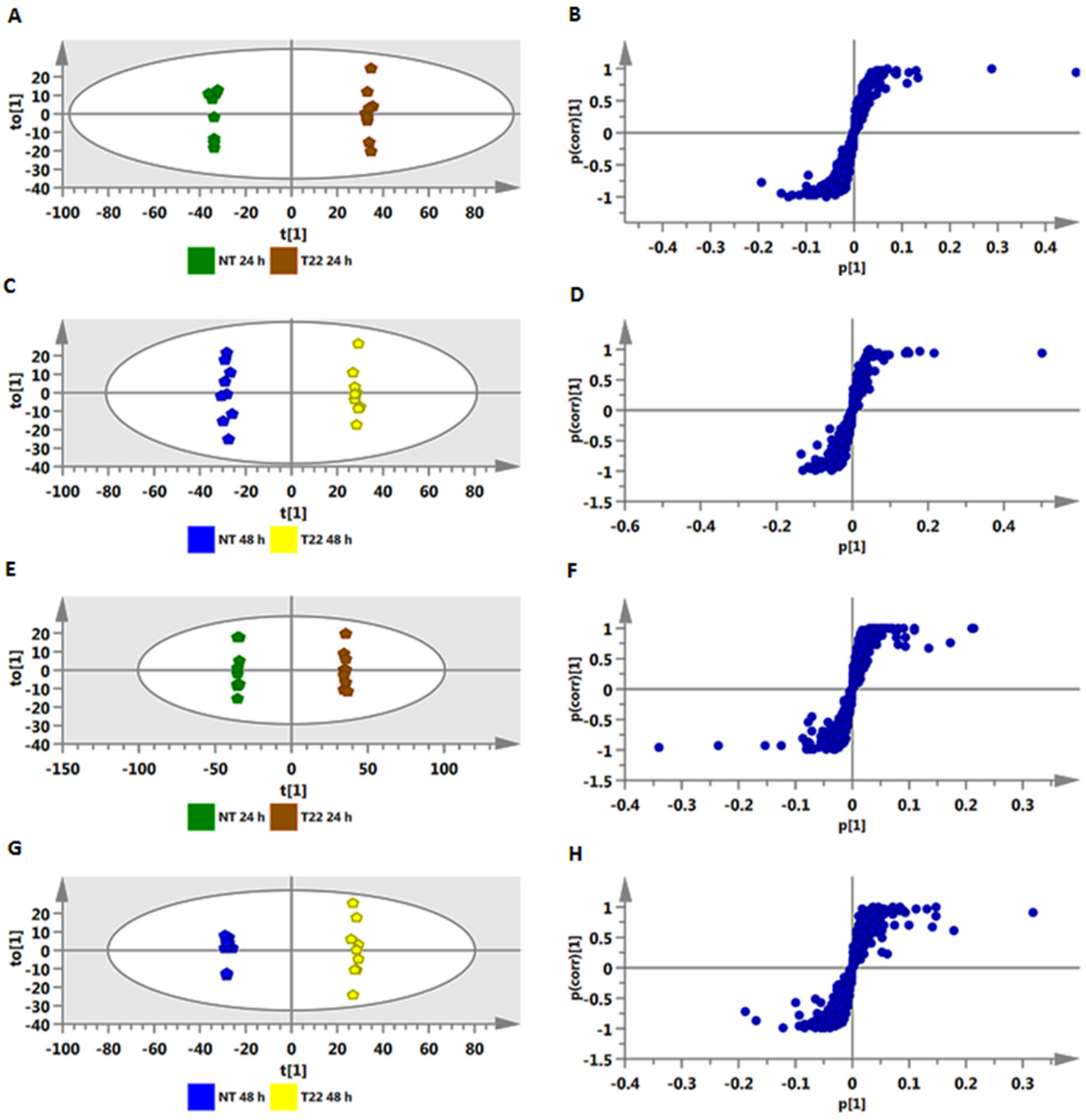

Figure S17. Identification of discriminatory biomarkers of *Paenibacillus alvei* T22-treated leaf samples using OPLS-DA modeling. The OPLS-DA score plots (**A, C, E** and **G**) and the corresponding S-plots (**B, D, F** and **H**) show the different clustering of treated and non-treated samples. The ellipse represents Hotelling's T^2 with 95% confidence. Model validation by CV-ANOVA showed high model significance with a p-value < 0.05. (**A – D**) = ESI negative data and (**E – H**) = ESI positive data.

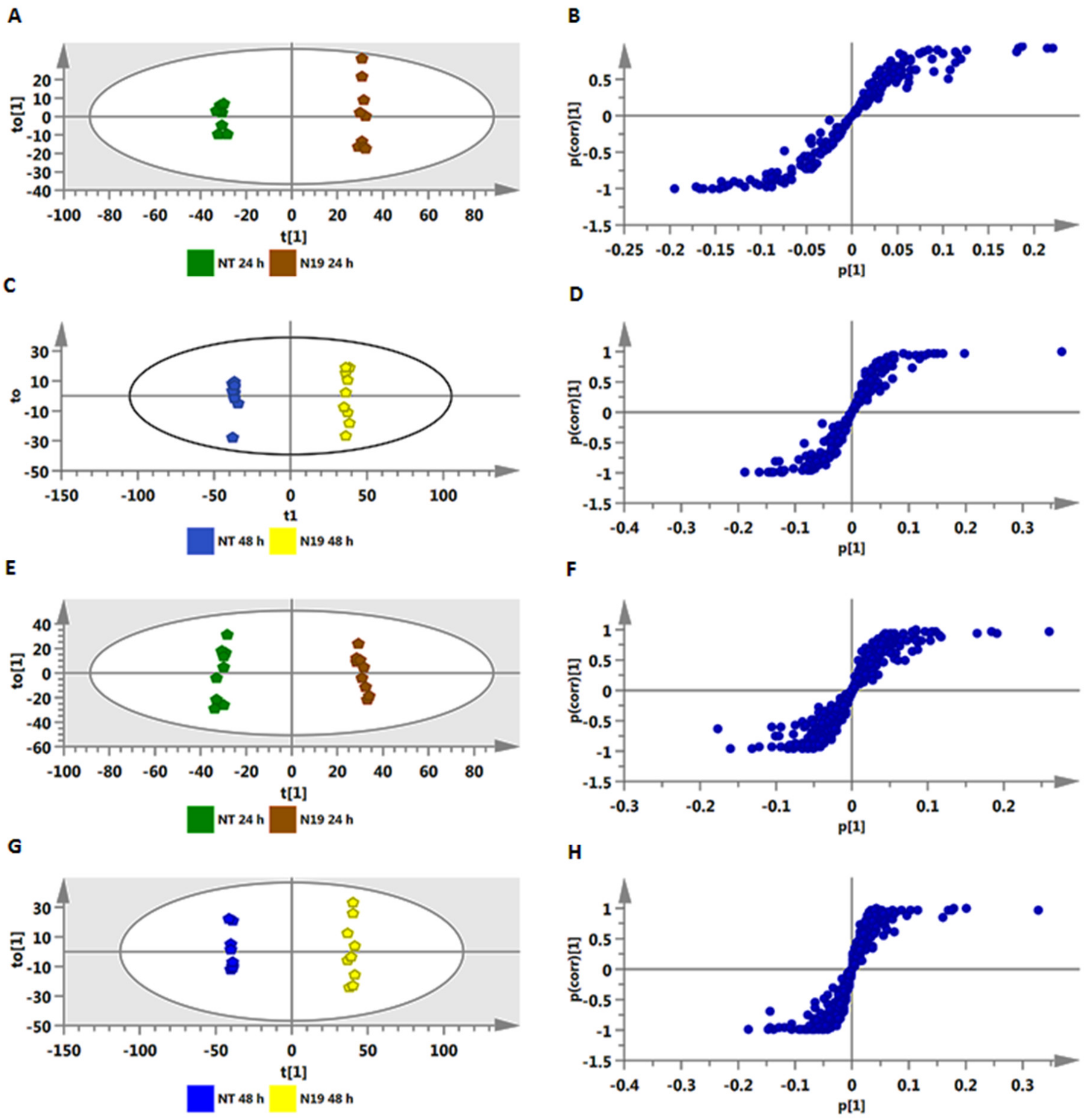

Figure S18. Identification of discriminatory biomarkers of *Pseudomonas koreensis* N19-treated stem samples using OPLS-DA modeling. The OPLS-DA score plots (**A, C, E** and **G**) and the corresponding S-plots (**B, D, F** and **H**) show the different clustering of treated and non-treated samples. The ellipse represents Hotelling's T^2 with 95% confidence. Model validation by CV-ANOVA showed high model significance with a *p*-value < 0.05. (**A** – **D**) = ESI negative data and (**E** – **D**) = ESI positive data.

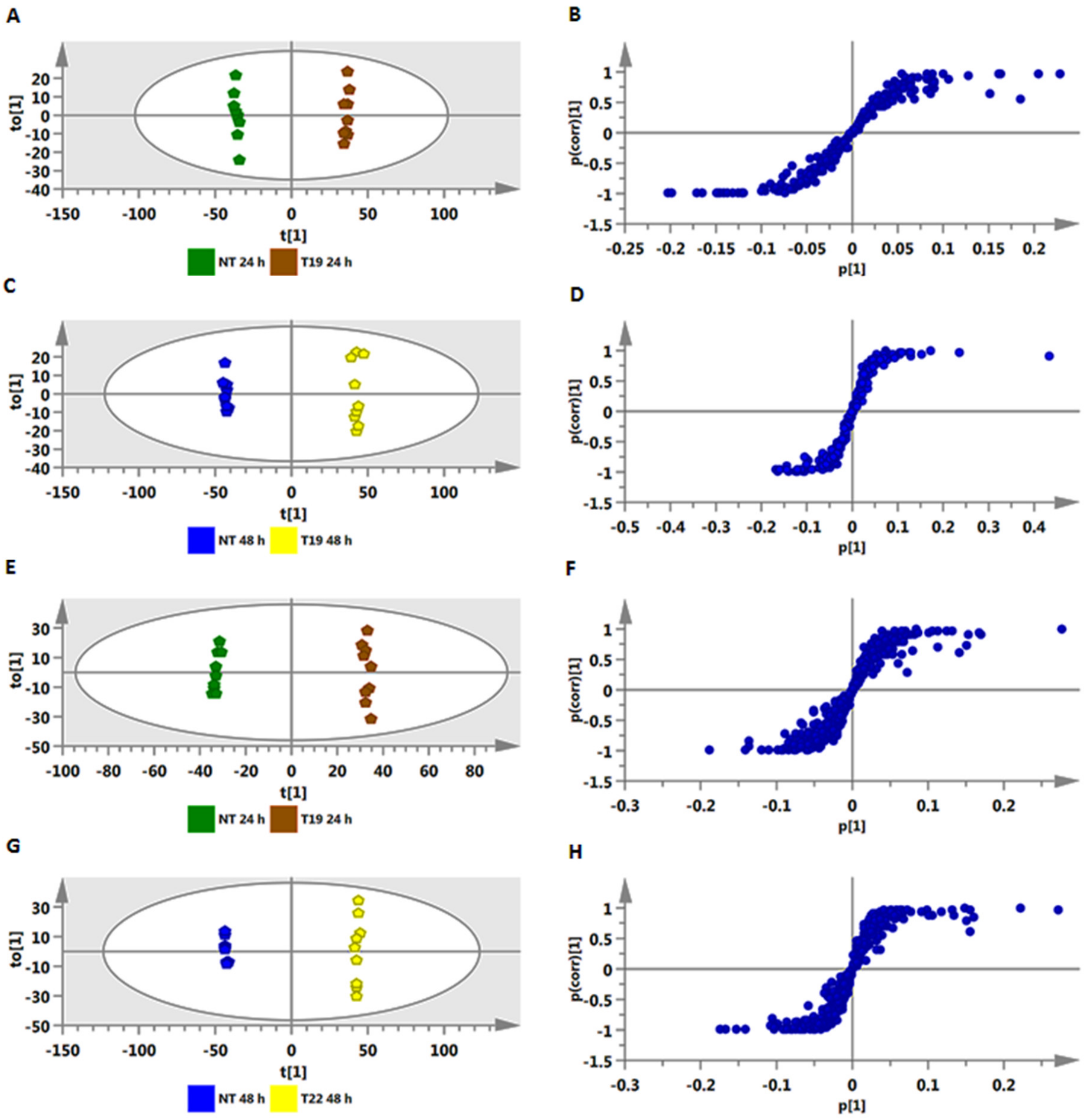

Figure S19. Identification of discriminatory biomarkers of *Lysinibacillus sphaericus* T19-treated stem samples using OPLS-DA modeling. The OPLS-DA score plots (**A, C, E** and **G**) and the corresponding S-plots (**B, D, F** and **H**) show the different clustering of treated and non-treated samples. The ellipse represents Hotelling's T^2 with 95% confidence. Model validation by CV-ANOVA showed high model significance with a p-value < 0.05. (**A** – **D**) = ESI negative data and (**E** – **D**) = ESI positive data.

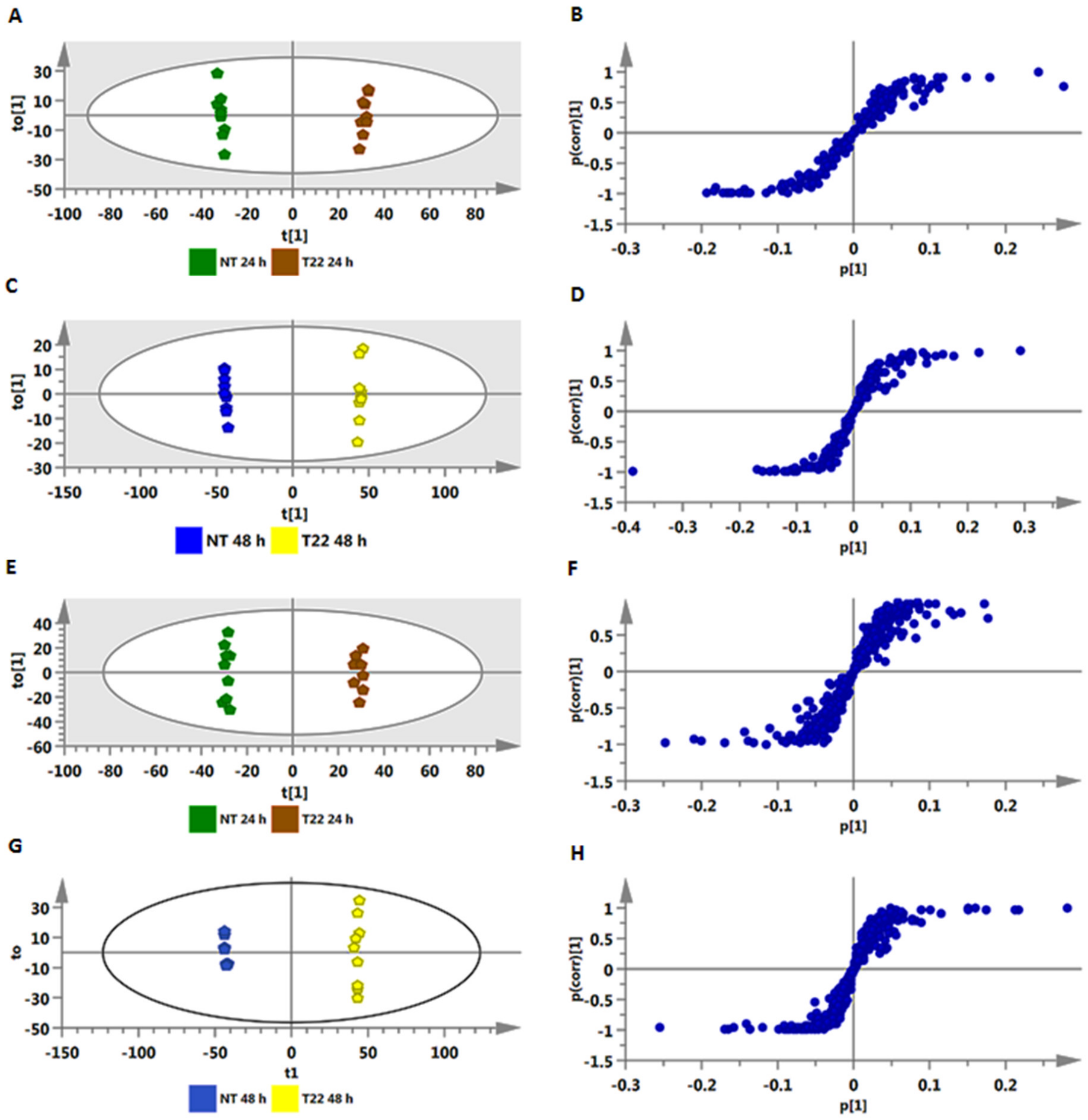

Figure S20. Identification of discriminatory biomarkers of *Paenibacillus alvei* T22-treated stem samples using OPLS-DA modeling. The OPLS-DA score plots (**A, C, E** and **G**) and the corresponding S-plots (**B, D, F** and **H**) show the different clustering of treated and non-treated samples. The ellipse represents Hotelling's T^2 with 95% confidence. Model validation by CV-ANOVA showed high model significance with a p-value < 0.05. (**A** – **D**) = ESI negative data and (**E** – **D**) = ESI positive data.

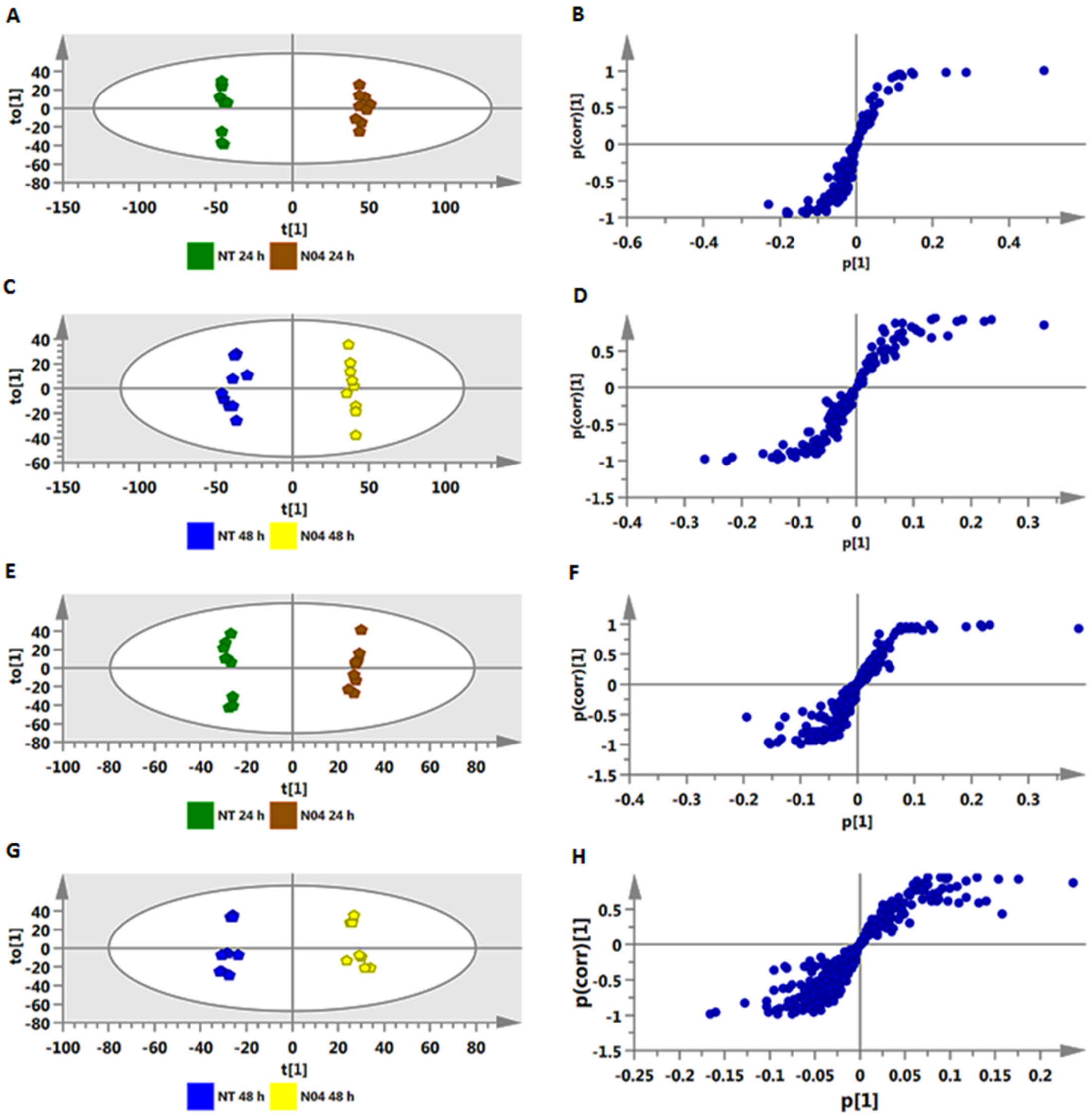

Figure S21. Identification of discriminatory biomarkers of *Pseudomonas fluorescens* N04-treated root samples using OPLS-DA modeling. The OPLS-DA score plots (**A, C, E** and **G**) and the corresponding S-plots (**B, D, F** and **H**) show the different clustering of treated and non-treated samples. The ellipse represents Hotelling's T^2 with 95% confidence. Model validation by CV-ANOVA showed high model significance with a *p*-value < 0.05. (**A** – **D**) = ESI negative data and (**E** – **D**) = ESI positive data.

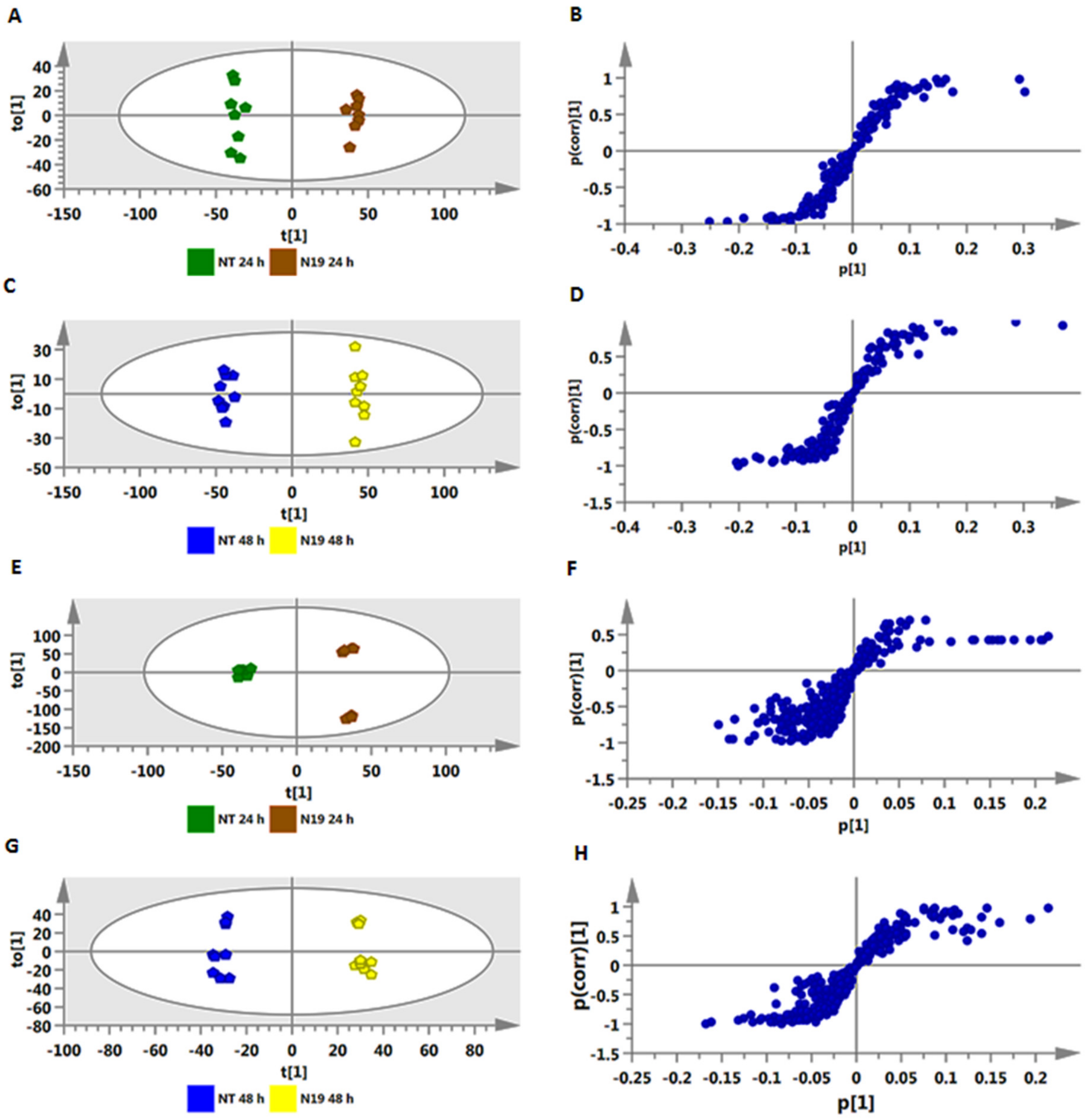

Figure S22. Identification of discriminatory biomarkers of *Pseudomonas koreensis* N19-treated root samples using OPLS-DA modeling. The OPLS-DA score plots (**A, C, E** and **G**) and the corresponding S-plots (**B, D, F** and **H**) show the different clustering of treated and non-treated samples. The ellipse represents Hotelling's T^2 with 95% confidence. Model validation by CV-ANOVA showed high model significance with a *p*-value < 0.05. (**A** – **D**) = ESI negative data and (**E** – **D**) = ESI positive data.

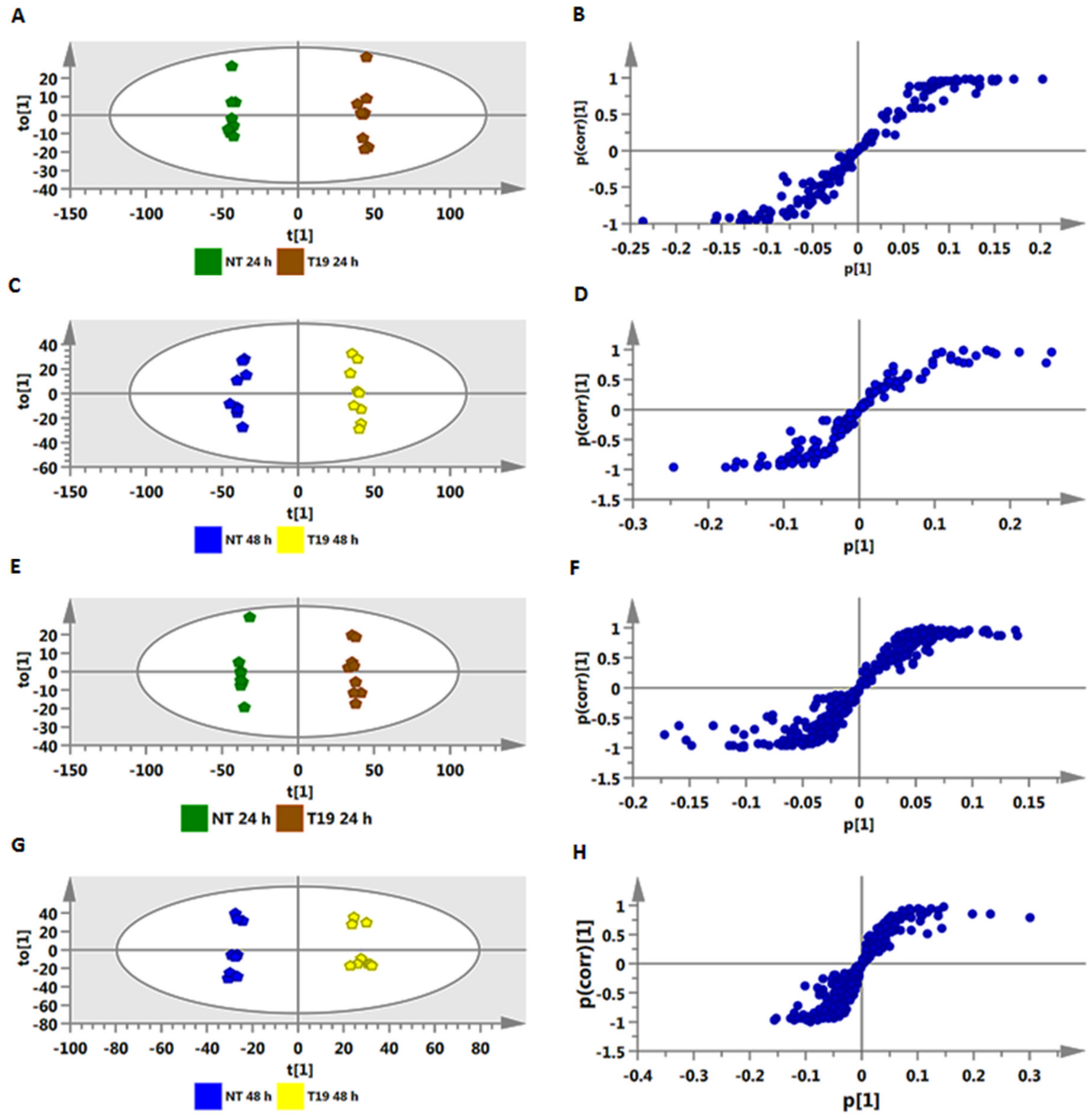

Figure S23. Identification of discriminatory biomarkers of *Lysinibacillus sphaericus* T19-treated root samples using OPLS-DA modeling. The OPLS-DA score plots (**A, C, E** and **G**) and the corresponding S-plots (**B, D, F** and **H**) show the different clustering of treated and non-treated samples. The ellipse represents Hotelling's T^2 with 95% confidence. Model validation by CV-ANOVA showed high model significance with a *p*-value < 0.05. (**A** – **D**) = ESI negative data and (**E** – **D**) = ESI positive data.

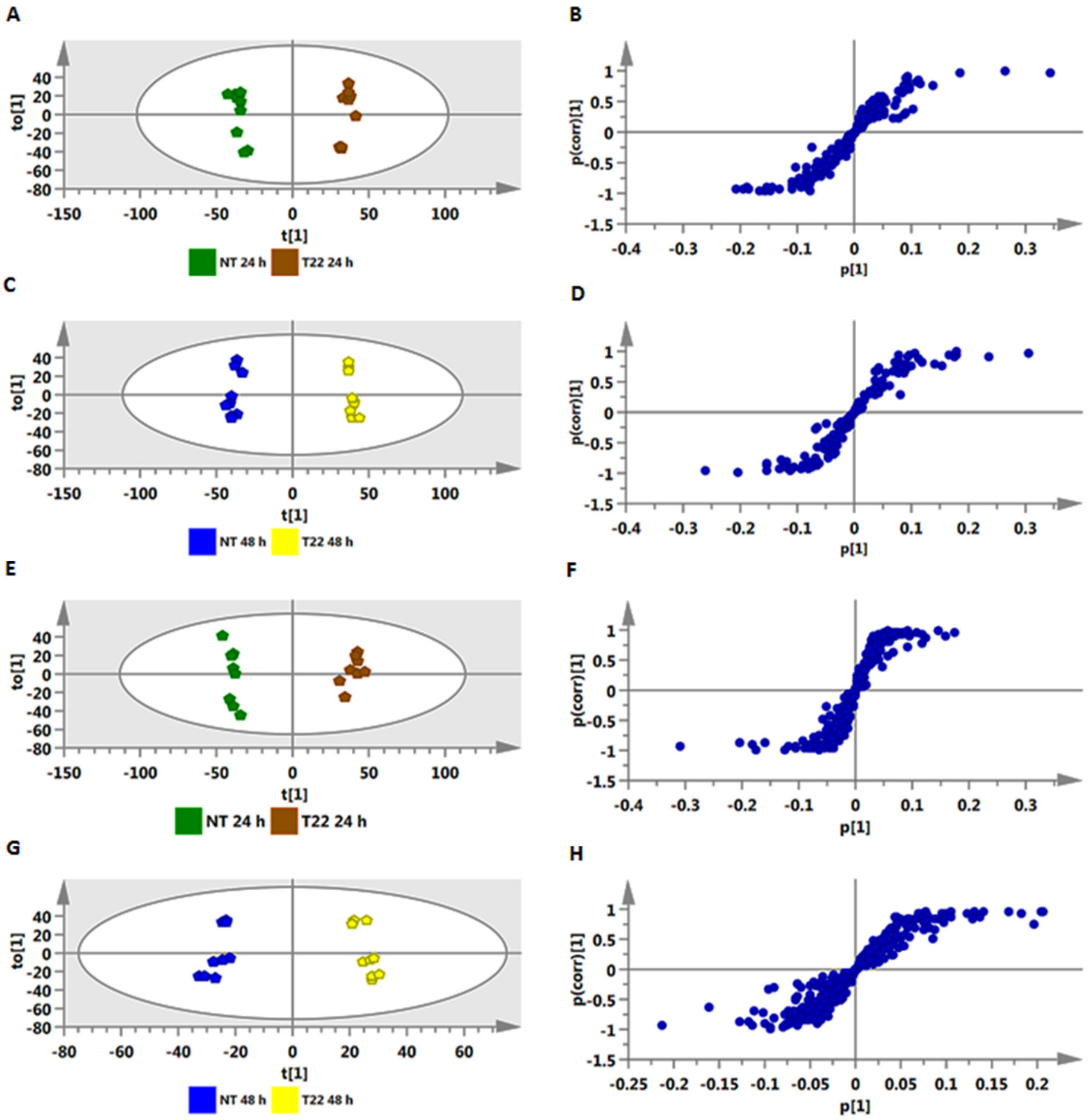

Figure S24. Identification of discriminatory biomarkers of *Paenibacillus alvei* T22-treated root samples using OPLS-DA modeling. The OPLS-DA score plots (**A, C, E** and **G**) and the corresponding S-plots (**B, D, F** and **H**) show the different clustering of treated and non-treated samples. The ellipse represents Hotelling's T^2 with 95% confidence. Model validation by CV-ANOVA showed high model significance with a *p*-value < 0.05. (**A** – **D**) = ESI negative data and (**E** – **D**) = ESI positive data.

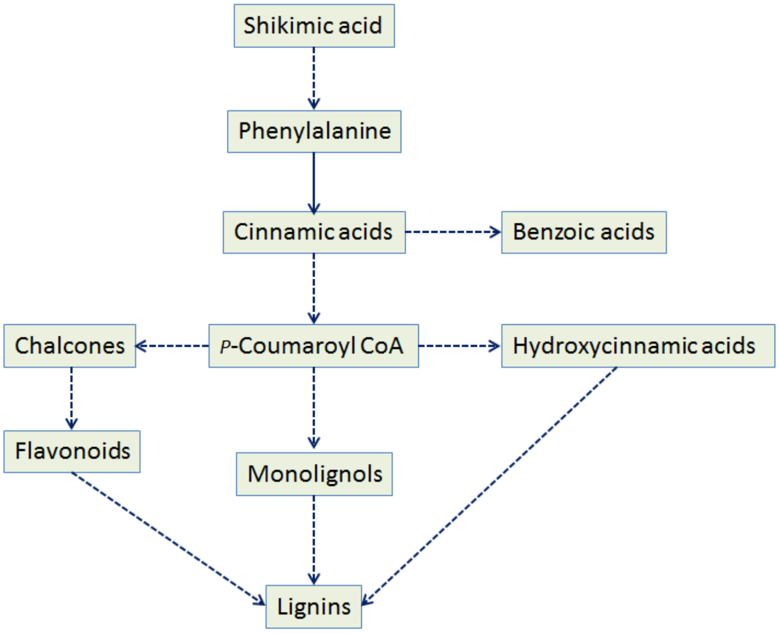

Figure S25. Schematic overview of branch pathways of the phenylpropanoid pathway leading to the synthesis of benzoic acids, hydroxycinnamic acid derivatives and flavonoids. Dashed arrows represent multiple biosynthetic reactions.
